# Association of polyalanine and polyglutamine coiled coils mediates expansion disease-related protein aggregation and dysfunction

**DOI:** 10.1093/hmg/ddu049

**Published:** 2014-02-04

**Authors:** Ilaria Pelassa, Davide Corà, Federico Cesano, Francisco J. Monje, Pier Giorgio Montarolo, Ferdinando Fiumara

**Affiliations:** 1 Department of Neuroscience and; 2 Department of Chemistry, University of Torino, Torino 10125, Italy; 3 Center for Molecular Systems Biology, University of Torino, Torino 10123, Italy; 4 Department of Neurophysiology and Neuropharmacology,Medical University of Vienna, Vienna 1090, Austria; 5 National Institute of Neuroscience (INN), Torino 10125, Italy

## Abstract

The expansion of homopolymeric glutamine (polyQ) or alanine (polyA) repeats in certain proteins owing to genetic mutations induces protein aggregation and toxicity, causing at least 18 human diseases. PolyQ and polyA repeats can also associate in the same proteins, but the general extent of their association in proteomes is unknown. Furthermore, the structural mechanisms by which their expansion causes disease are not well understood, and these repeats are generally thought to misfold upon expansion into aggregation-prone β-sheet structures like amyloids. However, recent evidence indicates a critical role for coiled-coil (CC) structures in triggering aggregation and toxicity of polyQ-expanded proteins, raising the possibility that polyA repeats may as well form these structures, by themselves or in association with polyQ. We found through bioinformatics screenings that polyA, polyQ and polyQA repeats have a phylogenetically graded association in human and non-human proteomes and associate/overlap with CC domains. Circular dichroism and cross-linking experiments revealed that polyA repeats can form—alone or with polyQ and polyQA—CC structures that increase in stability with polyA length, forming higher-order multimers and polymers *in vitro*. Using structure-guided mutagenesis, we studied the relevance of polyA CCs to the *in vivo* aggregation and toxicity of RUNX2—a polyQ/polyA protein associated with cleidocranial dysplasia upon polyA expansion—and found that the stability of its polyQ/polyA CC controls its aggregation, localization and toxicity. These findings indicate that, like polyQ, polyA repeats form CC structures that can trigger protein aggregation and toxicity upon expansion in human genetic diseases.

## INTRODUCTION

Proteins often contain amino acid homopolymeric stretches of variable length, from a few up to tens of units (1). Those formed by glutamine (Q) and alanine (A) have been more extensively studied owing to their involvement in at least 18 human genetic diseases, including Huntington's disease and cleidocranial dysplasia (2,3). In these disorders, as a consequence of triplet-expansion genetic mutations, polyQ and polyA stretches in certain proteins expand beyond a critical length, assuming an aggregation-prone conformation that leads to the buildup of intracellular protein aggregates and to cellular toxicity (4).

Interestingly, polyQ and polyA stretches can co-occur in the same proteins, sometimes in contiguous or quasi-contiguous position, even in proteins associated with polyQ- or polyA-expansion diseases such as Ataxin-7 (ATX7) and Runt-related transcription factor 2 (RUNX2) (5,6). Moreover, alternate glutamine-alanine (QA) repeats of variable length (polyQA) have also been occasionally observed, either alone or in association with pure polyQ and/or polyA stretches (7,8). The co-occurrence of repeats of different amino acids has been noticed in eukaryotic proteins of different species (9–12). However, a systematic phylogenetic analysis of the occurrence as well as of the structural and functional meaning of the association between polyQ, polyA and polyQA repeats in proteomes is still lacking.

The structure of polyQ and polyA stretches has been the subject of extensive research, because it is considered the key determinant of the molecular pathogenesis of polyQ- and polyA-expansion diseases (4), in association with emerging RNA-mediated mechanisms (13). However, owing to their insolubility and aggregation proneness, the atomic-level structure of full-length polyQ- or polyA-expanded proteins has not been defined yet. PolyQ and polyA stretches are generally thought to have a tendency to misfold into β-sheets when they exceed a critical length, a transition held as underlying their aggregation and toxicity, in analogy with some well-characterized amyloids known to form β-sheet-based aggregates (3,14). However, recent studies have revealed different structural propensities and a greater complexity in the structural dynamics of polyQ proteins (4). In particular, we have recently found that polyQ stretches associate and overlap with coiled-coil (CC) super-secondary structures, which are also enriched in the interactomes of polyQ proteins (15). Among other functions, CC structures mediate protein–protein interactions, and protein oligo-/poly-merization (16). Indeed, we have found that CCs can trigger aggregation and mediate the toxicity of polyQ-expanded proteins such as huntingtin (15). These findings revealed a critical role for CC structures in the molecular pathogenesis of polyQ-expansion diseases and have been further confirmed and extended to other polyQ and Q-rich proteins (17–22). The association, and at times even contiguity, of CC-prone polyQ repeats with polyA and polyQA repeats raises the question whether also these latter repeats can be involved in the assembly of CC structures, thus suggesting the possibility that polyQ and polyA stretches might share a common CC structural propensity and, upon expansion, cause toxicity and disease through a unitary CC-based mechanism. Indeed, experimental evidence indicates that polyA stretches have a marked helical propensity (23,24), and early studies have even used the polyA helix structure in idealized CC models (25). However, the actual potential of polyA stretches to form CC-structures—either alone or in association with polyQ repeats—as well as the possible role of CCs in the molecular pathogenesis of polyA-expansion diseases is yet to be determined.

To address these issues, we have first undertaken a systematic bioinformatics screening of human and non-human proteomes in order to define the degree of co-occurrence in proteins of polyQ, polyA and polyQA stretches, and their degree of association/overlap with CC domains. The results of our analysis indicated a close, phylogenetically graded association between polyQ, polyA and polyQA stretches, as well as a significant association/overlap of these repeats with CC structures. Based on these findings, through a combination of biophysical, biochemical and cell biological approaches, we have studied both *in vitro* and *in vivo* the actual CC propensity of polyA stretches of variable length, their oligomeric states and their role in intracellular protein aggregation and toxicity. Our results indicate that polyA stretches, either alone or in association with polyQ and polyQA repeats, can form CC structures and that these structures have a critical role for the aggregation and toxicity of mutant forms of RUNX2, the molecular mediator of cleidocranial dysplasia upon polyA expansion.

## RESULTS

### Phylogenetically graded association of polyQ and polyA repeats in eukaryotic proteomes

**Figure 1.**
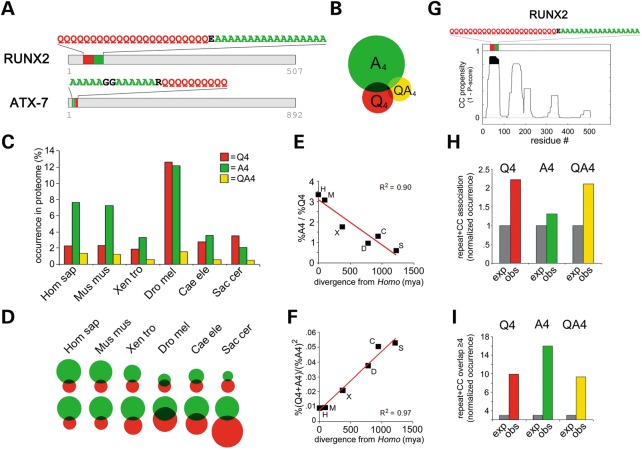
Phylogenetically graded association of polyQ, polyA and polyQA repeats. (**A**) Schematic representation of the primary sequence (gray bars) of the human triplet-expansion disease proteins RUNX2 and ATX7. PolyQ and polyA stretches are highlighted in red and green, respectively, and their sequence is reported earlier above each bar. (**B**) Proportional Venn diagram showing the relative occurrence of Q4 (red), A4 (green) and QA4 (yellow) proteins in the human proteome. Overlap shaded areas indicate Q4 + A4, Q4 + QA4, A4 + QA4 and Q4 + A4 + QA4 proteins. (**C**) Histogram showing the percent occurrence of Q4, A4 and QA4 proteins in human and non-human proteomes. (**D**) Graphically normalized Venn diagrams showing the relative proportions of Q4 (red), A4 (green) and Q4 + A4 proteins (overlap area) in human and non-human proteomes. Upper row: the size of red circles (Q4) is scaled to the human Q4 circle. Lower row: the size of green circles (A4) is scaled to the human A4 circle. Note the progressive increase in the A4/Q4 ratio throughout phylogenesis. (**E**) Correlation of the %A4/%Q4 ratio in *Homo* (H), *Mus* (M), *Xenopus* (X), *Drosophila* (D), *Caenorhabditis* (C) and *Saccharomyces* (S) with phylogenetic distances, estimated as the time of evolutionary divergence of each species from *Homo sapiens* expressed in millions of years (*mya*). (**F**) For each proteome, the co-occurrence of Q4 and A4 repeats in proteins is expressed as the proportion of polyA proteins containing a polyQ stretch (i.e. %(Q4 + A4)/%A4) normalized to the percentage of A4 proteins (i.e. %(Q4 + A4)/%A4^2^). This normalized overlap decreases regularly from *Saccharomyces* to *Homo*, correlating with phylogenetic distances (Pearson's *r* = 0.98, *P* < 0.001, with a *R*^2^ = 0.97). (**G**) Per-residue CC prediction plot for RUNX2 obtained using Paircoil2. Peaks indicating regions with high (0.8–1) CC propensity (1 minus *P*-score) are highlighted in black. Colored bars indicate polyQ (red) and polyA (green) stretches, and the corresponding primary sequence is reported. (**H**) Histogram displaying the observed (obs) and expected (exp) occurrence of CC + Q4, CC + A4 and CC + QA4 proteins in the human proteome. In each case, the observed values are normalized to the expected ones. The observed CC + repeat associations in the human proteome range between 1.3 and 2.2 times more than expected based on the respective percentages of Q4, A4, QA4 and of predicted CC proteins in the whole proteome (*P* < 0.05, χ^2^ test, in all instances). (**I**) Histogram displaying the observed (obs) and expected (exp) number of proteins with a CC/repeat overlap of at least four residues in the human proteome. In each case, the observed values are normalized to the expected ones. The observed number of proteins with a CC/repeat overlap of at least four residues exceeds the values expected by chance from 10 to 16 times (*P* < 0.01, χ^2^ test, in all instances).

To study the occurrence of polyQ and polyA repeats throughout phylogenesis, and their co-occurrence as found in some expansion-disease-related proteins (e.g. ATX7 and RUNX2, Fig. 1A), we have undertaken a bioinformatics screening of the human and other five eukaryotic proteomes searching for proteins containing repeats of at least four Qs (‘Q4’), four As (‘A4’), or both. We found that the human proteome contains 2.29 and 7.73% of Q4 and A4 proteins, respectively (Fig. 1B and C). The percentages of polyQ and polyA proteins in the other proteomes seem to vary irrespective of phylogenetic relationships (Fig. 1C). Strikingly, however, the ratio between the percentage of polyA and polyQ proteins gradually increases from 0.58 in *Saccharomyces* to 3.37 in *Homo* (Fig. 1D and E), with a strong correlation with evolutionary distances, estimated as the time of evolutionary divergence of each species from *Homo sapiens* (26) (Pearson's *r* = −0.94, *P* < 0.01, *R*^2^ = 0.90). This indicates a phylogenetically graded, negative correlation in the occurrence of polyQ and polyA repeats in eukaryotic proteomes. We also noticed that QA tandem repeats (‘QA4’) are frequently found in Q4 and A4 proteins and that their occurrence is also negatively correlated with that of Q4 repeats throughout phylogenesis (Fig. 1B and C; Supplementary Material, Fig. S1A).

Next, we studied how the association of polyA and polyQ repeats varies throughout evolution (Fig. 1D and F). In all of the proteomes, we found a significant over-representation of proteins containing both Q and A repeats with respect to what is expected by chance (‘Q4 + A4’; *P* < 0.01, χ^2^ test; Supplementary Material, Figure S1B). QA4 repeats also significantly co-occur in the same proteins with Q or A repeats (*P* < 0.01 in both cases, *χ*^2^ test; Fig. 1B and C; Supplementary Material, Fig. S1B, Table S1). Again, the percentage of these proteins in proteomes changes in apparent independence of phylogenetic distances (Fig. 1C; Supplementary Material, Fig. S1C). However, there is a strong correlation between phylogenetic distances and the overlap between the polyA and polyQ protein groups, when values are normalized to the percentage of A4 proteins (Fig. 1F). Thus, proteomes with a higher percentage of polyA proteins have a higher proportion of these proteins containing also a polyQ stretch, and the more so the greater the phylogenetic distance from *Homo*.

Taken together, these observations show that polyQ, polyA and polyQA repeats occur and co-occur in eukaryotic proteomes in a phylogenetically graded manner from yeast to humans.

### Association and overlap of polyA and polyQA repeats with CC domains

PolyQ repeats frequently associate with CC domains in proteins and can participate themselves in the formation of CC super-secondary structures regulating the aggregation and toxicity of polyQ proteins (15,17). Considering the frequent co-occurrence of polyQ, polyA and polyQA repeats that was observed, we assessed whether polyA and polyQA repeats also associate with, and can form, CC structures either alone or in association with polyQ repeats. To this aim, we first systematically analyzed the CC propensity of Q4, A4 and QA4 proteins with Paircoil2 (27), an algorithm detecting CC heptad repeats in protein sequences (Fig. 1G–I). We found a significant over-representation in human and non-human proteomes of proteins containing both CC domains and A4 repeats (‘CC + A4’), as well as of proteins containing both CC and Q4 (‘CC + Q4’) or QA4 (‘CC + QA4’) repeats (Fig. 1H). Furthermore, there is a significant degree of overlap between predicted CC domains and homopolymeric A, Q or QA repeats (Fig. 1I), as exemplified by the RUNX2 protein (Fig. 1G). Similar results were also found for the other five non-human proteomes (not shown).

These CC proteins revealed a striking variety of repeat combinations, often with multiple short polyQ, polyA and polyQA segments within the same CC domain (Supplementary Material, Fig. S1D). These polyQ, polyA and polyQA segments can be flanked/interrupted by CC-stabilizing hydrophobic residues often with heptad spacing in positions *a/d*, and by charged residues in positions *e/g*, in a variety of possible arrangements including leucine and valine zippers (28) (Supplementary Material, Fig. S1E).

These findings strongly indicate the possibility that A and QA repeats may be involved in the formation of CC structures similarly to Q repeats and that the variable number and arrangement of Q, A and QA stretches in these complex CCs may determine their biological properties.

### PolyA peptides form CC structures that are stabilized upon polyA elongation

**Figure 2.**
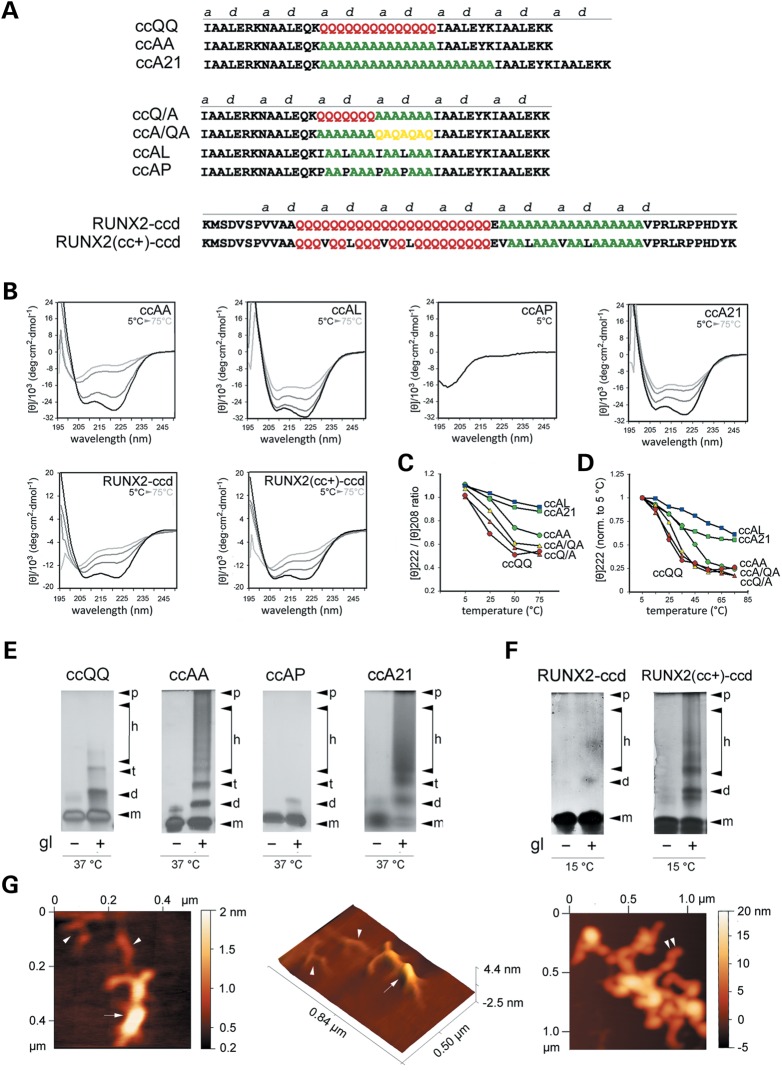
Structure and oligo-/poly-merization of polyQ, polyA, polyQA and mixed CC peptides. (**A**) Primary sequence of polyQ, polyA, polyQA and mixed CC peptides. The CC heptad register is shown, highlighting positions *a* and *d*. Some polyA peptides are stabilized by hydrophobic residues or destabilized by proline residues in positions *a*/*d*. Two peptides are derived from the RUNX2 protein sequence from K24 to D84, with [RUNX2(cc+)-ccd], or without (RUNX2-ccd) CC-stabilizing mutations. The YK di-peptide has been added for the spectrophotometric measurement of concentration (Y) and to improve solubility (K). (**B**) Circular dichroism spectra of some of the peptides shown in A, dissolved in benign buffer, pH 7.4. Spectra were collected at different temperatures, starting from 5°C (black traces) to 25°C (dark gray traces) and 50°C (medium gray traces) up to 75°C (light gray traces). (**C**) Quantification of the 222-/208-nm ellipticity ratio as a function of temperature, based on the CD spectra shown in B (blue squares, ccAL; green squares, ccA21; green circles, ccAA; yellow triangles, ccA/QA; orange triangle, ccQ/A; red circles, ccQQ). (**D**) Quantification of the 222-nm ellipticity as a function of temperature in the 5–75°C range. For each peptide, values are normalized to the 222-nm ellipticity measured at 5°C. Labeling as in C. (**E** and **F**) Silver-stained Tricine–SDS electrophoretic gels separating chemically cross-linked peptides ccQQ, ccAA, ccAP and ccA21 (E) or RUNX2-ccd and RUNX2(cc+)-ccd (F). Peptides were cross-linked by incubation with glutaraldehyde (*gl+*) for 15 min at 37 or 15°C, to better highlight differences between them in suitable temperature ranges, based on their thermal stability as determined in CD experiments. In control lanes, peptides that were not cross-linked (*gl−*) run essentially as monomers (*m*). Cross-linked peptides show the variable presence of dimers (*d*), higher-order multimers (*h*) and eventually polymers (*p*). (**G**) AFM images of structures formed by the peptide ccA21. The middle panel represents a three-dimensional rendering of the image shown in the left panel. Arrowheads indicate elementary fibrils and arrows indicate the higher-order assemblies of these fibrils. The right panel shows a tangle of larger fibers. The extremity of one of these is indicated by the double arrowhead.

To determine whether polyA stretches, either alone or in association with polyQ and polyQA repeats, can indeed be part of CC structures, we synthesized a series of peptides (Fig. 2A) based on a four-heptad model peptide (29), that was previously employed to characterize the CC propensity of polyQ stretches (15), and studied them with circular dichroism (CD) spectropolarimetry to define their secondary structure and CC propensity. In particular, we inserted at the core of this model peptide polyA stretches of different length (i.e. 14 alanines in peptide ccAA and 21 alanines in peptide A21) or polyA stretches in combination with polyQ and polyQA repeats (peptides ccQ/A and ccA/QA). We also generated variants of peptide ccAA in which the CC propensity was enhanced by the insertion of CC-stabilizing hydrophobic residues (peptide ccAL) or diminished by CC-disrupting prolines (peptide ccAP) in *a/d* heptad positions. As a reference control, we also synthesized the ccQQ peptide, containing 14 Qs, whose CC propensity and stability were previously characterized (15). Furthermore, we synthesized two peptides encompassing the polyQ/polyA coiled-coil domain (ccd) of RUNX2. One peptide (RUNX2-ccd) contains the wild-type (wt) sequence, whereas the other peptide, RUNX2(cc+)-ccd, contains the same ccd with CC-stabilizing mutations in *a/d* heptad positions similar to those used for the ccAL peptide. In fact, some As and Qs were replaced by hydrophobic residues such as leucine and valine. Valine was chosen because this amino acid is already present in *a/d* heptad positions adjacent to the polyQ/polyA region of RUNX2 (Supplementary Material, Fig. S1E). These peptides also contain a few RUNX2 residues adjacent to the polyA/polyQ CC that were included to help solubilize the peptides, given the presence of charged residues. The presence also of some scattered prolines in these flanking regions can limit their secondary structure stability, thus reducing their interference with the structural analysis of the polyQ/polyA core of these peptides.

Circular dichroism is widely used for studying the folding and stability of CCs. Distinctive signatures allow to discriminate between single and coiled helices, based on the ellipticity ratio at 222 and 208 nm (≥1 for CCs), the inversion of this ratio induced by trifluoroethanol (TFE), and the thermal stability of the folding (15,30–36).

The polyA peptide ccAA displayed CD spectra with minima at 208 and 222 nm, and a 222-/208-nm ratio ≥1 (1.10), indicating α-helical CC formation (Fig. 2B and C). As predictable for CC structures, the CC helical folding was considerably stabilized by the addition of canonical hydrophobic residues in position *a/d* of the polyA stretch (peptide ccAL, Fig. 2B and C) and completely disrupted by prolines at the same positions (peptide ccAP), as shown by a single minimum at ∼200 nm indicating a random coil conformation (Fig. 2B).

The presence of a longer polyA stretch (peptide ccA21) induced a further increase in the 222-nm ellipticity with a 222-/208-nm ratio ≥1, thus showing a greater degree of helical CC folding (Fig. 2B). Furthermore, the elongation of the polyA stretch conferred greater thermal stability to the helical CC folding of ccA21 with respect to ccAA, as observed by analyzing both the 222-nm ellipticity and the 222-/208-nm ratio at increasing temperatures (Fig. 2C and D). The helical CC signatures and thermal stability of both ccAA and ccA21 were more pronounced than those of ccQQ (Fig. 2B–D; Supplementary Material, Fig. S2A). As typical of CC structures, the presence of TFE brought the 222-/208-nm ratio of <1 without reducing the helical folding, indicating dissociation of the helices (Supplementary Material, Fig. S2B). Taken together, these findings indicate that polyA stretches can form α-helical CC structures that are even more stable that those formed by polyQ stretches and that the CC folding of polyA stretches can be modulated in opposite directions by appropriate CC-stabilizing or -destabilizing substitutions. Furthermore, these results also indicate that the elongation of polyA repeats leads to an increased CC stability, as shown by the similar behavior of ccA21 and ccAL peptides.

Next, we studied the two peptides combining polyA with polyQ or polyQA stretches (peptides ccQ/A and ccA/QA, respectively). Interestingly, both displayed CC features, with a degree of stability that was intermediate between peptides with pure polyA or polyQ stretches (ccAA>ccA/QA>ccQ/A>ccQQ; Fig. 2C–D; Supplementary Material, Fig. S2A), in apparent correlation with the relative proportion of A and Q residues in *a/d* (4:0 > 3:1 > 2:2 > 0:4). These observations indicate that the variable combination of polyQ, polyA and polyQA stretches within CCs can finely tune their stability and therefore their biological properties.

Finally, we analyzed the RUNX2 peptides. In agreement with the predictions, RUNX2-ccd displayed clear α-helical features, with minima at 222 and 208 nm, and a 222-/208-nm elipticity ratio of ≥1 (1.01) indicative of CC formation (Fig. 2B). RUNX2(cc+)-ccd displayed similar features and was more stable in thermal denaturation experiments (Fig. 2B; Supplementary Material, Fig. S2C), consistent with the presence of CC-stabilizing substitutions in its polyQ/polyA stretch. Notably, about two-thirds of the amino acid sequence of these two peptides are made of polyQ and polyA stretches (23Q + 17A) separated only by a single glutamate residue. The sequences flanking these stretches contain scattered proline residues that would disrupt the CC structure in these flanking sequences (see Figs 1G and 2A). The fact that this peptide still exhibits CC features strongly indicates that the polyQ and polyA stretches at the core of this peptide are primarily responsible for the formation of the CC structure. Furthermore, as typical of CCs, the structure of both peptides was sensitive to TFE (e.g. Supplementary Material, Fig. S2D), which lowered the 222-/208-nm elipticity ratio and enhanced at the same time the alpha-helical folding. These findings strongly corroborate the prediction that the polyQ/polyA stretch of RUNX2 can form CCs, and, together with the results obtained with model peptides, indicate that mutations within polyQ/polyA stretches such as those we employed can effectively enhance or disrupt the CC structure.

### PolyA CC peptides form higher-order multimers and polymers *in vitro*

CC helical assemblies can range from dimers to polymers. To assess the oligomeric state of the peptides studied with CD, we used chemical cross-linking with glutaraldehyde (Fig. 2E and F; Supplementary Material, Fig. S2E) and found that polyA-containing peptides can variably populate dimeric and higher-order multimeric states, up to polymers. Peptide ccAA formed in fact dimers, higher-order oligomers/multimers and even polymers, whereas ccQQ under the same conditions formed only lower-order oligomers and ccAP was essentially monomeric, in very good agreement with the CC propensity and stability of each peptide as shown by the CD experiments (Fig. 2E). This correlation was further confirmed by studying peptides with a longer polyA tract or with mixed polyA/polyQ or /polyQA tracts. In fact, as for the CD experiments, ccQ/A and ccA/QA displayed intermediate features between ccQQ and ccAA (Supplementary Material, Fig. S2E), thus indicating that the multimerization tendency of polyA CCs can be finely modulated by their combination with polyQ and polyQA segments. The ccA21 peptide showed an even higher tendency than ccAA to form multimers and polymers (Fig. 2E) that may drive aggregation *in vivo*. Next, we analyzed the oligomeric state of the RUNX2 peptides (Fig. 2F). Chemical cross-linking showed that RUNX2-ccd forms dimers and some lower-order oligomers, whereas RUNX2(cc+)-ccd forms, besides dimers, also higher-order multimers and polymers. These observations are in good correlation with the differential CC stability of the two peptides that was shown by the CD experiments, similar to what was observed for the CC model peptides.

Finally, we characterized the ultrastructural morphology of the polymeric assemblies formed by polyA CC peptides that were apparent in the chemical cross-linking experiments. To this aim, we performed an atomic force microscopy (AFM) analysis (37) of the ultrastructural organization of ccA21 polymers (Fig. 2G). CcA21 was chosen because it has a high polymerization tendency, based on the cross-linking experiments. AFM showed that this peptide forms fibrillar structures with a hierarchical organization by which elongated elementary fibrils (height <1 nm; Fig. 2G, left and middle panels), often branched, bundle to form progressively larger fibrils, reaching heights of >5 nm, that branch and intertwine forming complex tangles (Fig. 2G, right panel). Interestingly, similar profiles have been observed in previous AFM studies of both CC (38) and polyQ proteins (37). These findings show how polyA CC peptides can form fibrillar structures that organize into progressively larger bundles and tangles, which may represent the ultrastructural substrate for aggregate formation *in vivo*.

### Structure-guided mutagenesis of RUNX2, a polyQ/polyA protein associated with cleidocranial dysplasia upon polyA expansion

**Figure 3.**
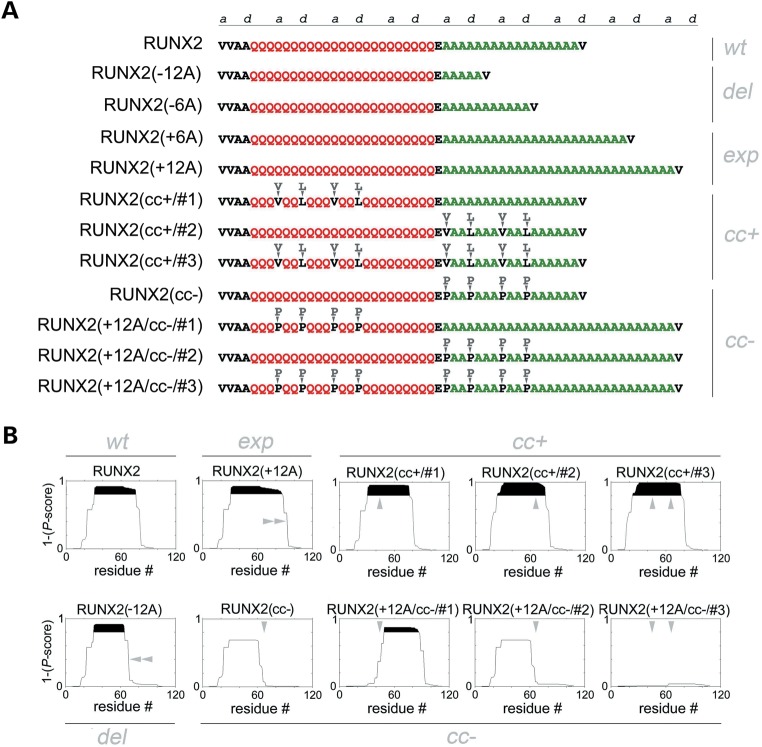
Structure-guided mutagenesis of wt and polyA-expanded RUNX2. (**A**) Design of polyQ and polyA mutants of RUNX2. For simplicity, only part of the primary sequence, corresponding to the fragment between V31 and V76 of wt RUNX2, and between V31 and V88 of RUNX2(+12A), is shown. PolyA length variants (del, deletions and exp, expansions) are also shown. Substitutions with CC-stabilizing (*cc*+) and CC-destabilizing (*cc*−) amino acids are highlighted (arrowheads and letters) above each sequence. (**B**) CC propensity of RUNX2 mutants shown in A as determined by Paircoil2, expressed as 1 minus the *P*-score assigned to each amino acid in the primary sequence. Protein segments whose CC propensity is 0.8–1 are highlighted in black. For simplicity, only the prediction for the N-terminal part of RUNX2 is shown. The site and effect on CC propensity of the different polyA length variations (del, partial deletion and exp, expansion) and of the CC-stabilizing (*cc+*) and CC-destabilizing (*cc−*) mutations are highlighted by gray arrowheads. Vertical arrowheads indicate the site and effect of CC-stabilizing and CC-destabilizing mutations. Horizontal arrowheads indicate deletion or expansions of the predicted CC domain related to corresponding variations in polyA length.

To define the relevance of polyA CCs to the *in vivo* aggregation and toxicity of proteins involved in human polyA-expansion diseases, we focused on the transcription factor RUNX2 (Fig. 3; Supplementary Material, Fig. S3A), the molecular mediator of cleidocranial dysplasia upon polyA expansion. PolyA-expanded RUNX2 mislocalizes from the nucleus to the cytoplasm forming aggregates, causing ultimately cellular dysfunction. RUNX2 contains both polyQ and polyA stretches separated by a single glutamate residue, and this polyQ/polyA region is predicted to form a CC (Fig. 1G) that expands with polyA elongation up to 10–12 additional alanines (Fig. 3B) as observed in cleidocranial dysplasia patients (39,40). RUNX2 thus offers a particularly suitable model to explore the role of polyA CC structures, and their interplay with polyQ CCs, in the aggregation and dysfunction of a protein involved in human polyA-expansion disease.

To directly test *in vivo* the role of CC structures in the aggregation and dysfunction of RUNX2, we generated different mutant forms of polyA-expanded and non-expanded RUNX2 (Fig. 3A) with variably enhanced or reduced CC stability, as determined with Paircoil2 (Fig. 3B; Supplementary Material, Fig. S3A). We first generated polyA length variants of RUNX2 with +6A and +12A expansions, and −6A or −12A deletions. Then, we designed mutants of RUNX2 with enhanced (*cc+* mutants) or reduced (*cc−* mutants) CC propensity by using the same type of mutations that were able to effectively enhance or impair CC formation in RUNX2-ccd and in other CC peptides in the CD experiments (Fig. 2). We generated a set of mutants of non-expanded RUNX2 with enhanced CC propensity by inserting canonical CC-stabilizing hydrophobic residues (V and L) in positions *a/d* of two heptads within the CC heptad register established by the valine residues that immediately precede and follow the polyQ/polyA region (Fig. 3A; Supplementary Material, Fig. S1E). These stabilizing residues were introduced in the polyQ region (RUNX2/cc+/#1), in the polyA region (RUNX2/cc+/#2), or in both (RUNX2/cc+/#3) (Fig. 3A and B). We also generated a set of polyA-expanded mutants whose CC-propensity was disrupted by the insertion of proline residues in heptad positions *a/d* in the polyQ region [RUNX2(+12A/cc−/#1)], or in the polyA region [RUNX2(+12A/cc−/#2)], or in both [RUNX2(+12A/cc−/#3)] (Fig. 3A and B). A *cc−* mutant of non-expanded, wt RUNX2 was also generated [i.e. RUNX2(cc−); Fig. 3A and B].

### *In vivo* subcellular distribution and aggregation of RUNX2 are regulated by length and stability of its polyQ/polyA CC

**Figure 4.**
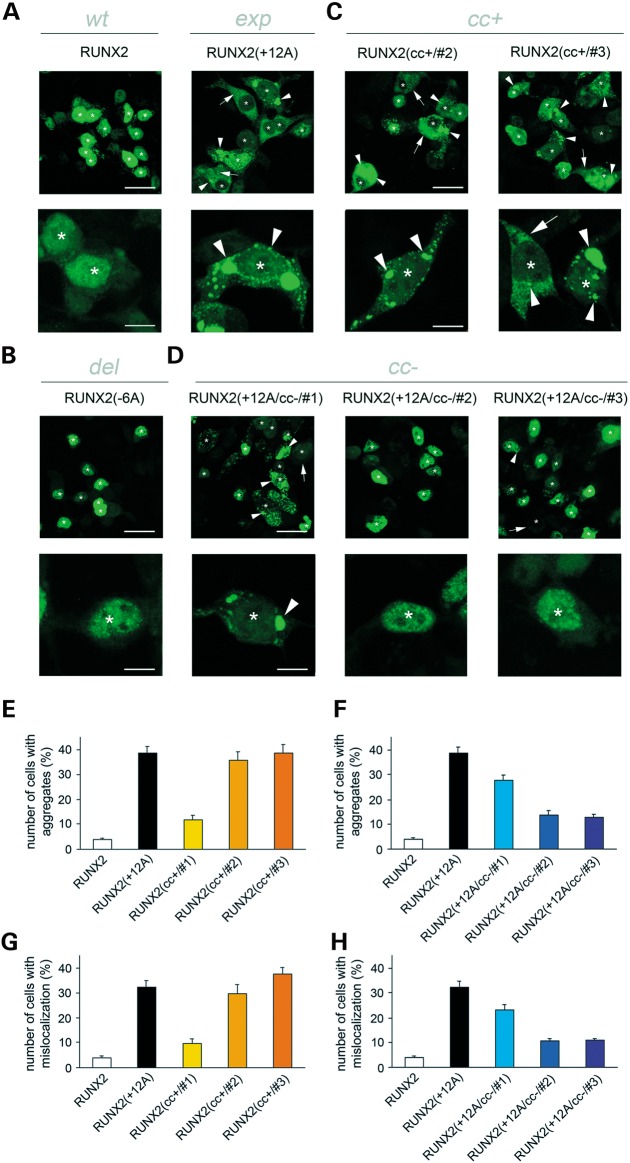
Subcellular distribution and aggregation of wt and mutant forms of RUNX2. (**A**) Confocal fluorescence imaging of HEK293 cells overexpressing for 72 h GFP-tagged wt RUNX2 (left panels) or polyA-expanded RUNX2 (right panels). Upper panels show representative 100 × 100 µm fields (calibration bar: 25 µm); lower panels show magnifications of one-two representative cells (calibration bar: 12 µm). Asterisks indicate cell nuclei. Arrows indicate cells with a cytoplasmic mislocalization of the overexpressed protein. Arrowheads indicate intracellular aggregates. (**B**) Imaging of cells expressing the RUNX2 mutant with partial deletion of the polyA stretch (−6A). Labeling and calibrations as in A. (**C**) Imaging of cells expressing RUNX2 mutants with CC-stabilizing mutations [*cc*+ mutants RUNX2(cc+/#2), and /#3]. Labeling and calibrations as in A. (**D**) Imaging of cells expressing polyA-expanded RUNX2 mutants with CC-destabilizing mutations [*cc−* mutants RUNX2(+12A/cc−/#1), /#2 and /#3]. Labeling and calibrations as in A. (**E**) Percentage of cells overexpressing for 72 h either GFP-tagged wt or polyA-expanded or *cc+* mutant RUNX2 that contain aggregates. (**F**) Percentage of cells overexpressing for 72 h GFP-tagged wt, polyA-expanded and *cc−* mutant RUNX2 that contain aggregates. (**G**) Percentage of cells overexpressing for 72 h GFP-tagged wt, polyA-expanded and *cc+* mutant RUNX2 in which the overexpressed protein shows cytoplasmic mislocalization. (**H**) Percentage of cells overexpressing for 72 h GFP-tagged wt, polyA-expanded and *cc-*mutant RUNX2 in which the overexpressed protein shows cytoplasmic mislocalization.

**Figure 5.**
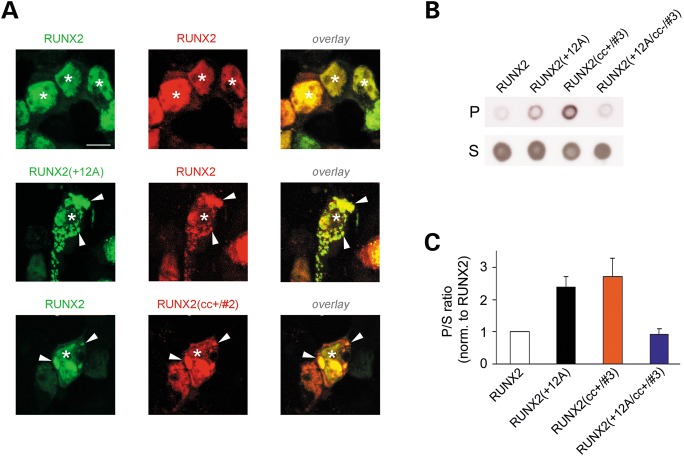
Recruitment of RUNX2 into aggregates and biochemical analysis of aggregation. (**A**) Confocal fluorescence imaging of HEK293 cells co-expressing for 72 h wt and mutant forms of RUNX2 tagged with either GFP or DsRed. Left column: GFP fluorescence images. Middle column: DsRed fluorescence images. Right column: overlay of GFP and DsRed fluorescence. Arrowheads indicate intracellular aggregates. Asterisks indicate nuclei. Upper row: cells co-expressing RUNX2-GFP and RUNX2-DsRed. The two proteins co-localize in the nucleus. Middle row: cells co-expressing RUNX2(+12A)-GFP and RUNX2-DsRed. RUNX2-DsRed is recruited into RUNX2(+12A)-GFP cytoplasmic aggregates. Lower row: cells co-expressing RUNX2-GFP and RUNX2(cc+/#2)-DsRed. RUNX2-GFP is recruited into aggregates of RUNX2(cc+/#2)-DsRed. Calibration bar: 10 µm. (**B**) Dot immunoblotting with anti-GFP antibodies of supernatant (S) and pellet (P) fractions of lysates of cells overexpressing for 72 h GFP-tagged RUNX2 or the indicated mutants of it. (**C**) Aggregation index (P/S ratio, see Methods) calculated from dot immunoblotting experiments for the indicated GFP-tagged constructs overexpressed for 72 h in HEK293 cells. Values are normalized to those of wt RUNX2.

To study the role of CC structures in the aggregation and localization of RUNX2 *in vivo*, we overexpressed GFP-tagged wt RUNX2 and its CC structural mutants in HEK293 cells and analyzed their subcellular distribution and aggregation state through confocal fluorescence microscopy and biochemical approaches (Figs 4 and 5). One-way ANOVA indicated overall significant differences in the occurrence of both aggregation and mislocalization of RUNX2 related to the expression of the different structural mutants [*F*(_7,80_) = 35.5, *P* < 0.001 and *F*_(7,80)_ = 30.2, *P* < 0.001, respectively].

Wild-type RUNX2 had an essentially nuclear localization in numerous subnuclear small foci (Fig. 4A, left panels), as observed previously (41). On the other hand, polyA-expanded RUNX2(+12A) (Fig. 4A, right panels, E and G) was often found to have a prevailing cytoplasmic localization (32.4 ± 0.2% of the cells, *n* = 14, 350 × 350 µm microscopy fields; *P* < 0.001 versus wt, Tukey HSD *post hoc* test), with one or more cytoplasmic or nuclear aggregates (38.6 ± 2.3% of the cells, *P* < 0.001 versus wt), consistent with previous studies of polyA-expanded forms of RUNX2 (42). Some cells displayed cytoplasmic aggregates but still had a predominantly diffuse nuclear localization, whereas others had a diffuse cytoplasmic distribution without aggregates. Conversely, a mutant with partial polyA deletion had the same nuclear localization of the wt form (Fig. 4B).

All the RUNX2 CC-enhancing mutants displayed some degrees of cytoplasmic mislocalization and aggregation, which were not significant when the CC-stabilizing mutations were restricted to the polyQ region [RUNX2(cc+/#1); Supplementary Material, Figs S3B; Fig. 4E and G] but were instead dramatic when the CC-stabilizing mutations were in the polyA stretch [RUNX2(cc+/#2) and /#3, Fig. 4C, E and G]. In fact, both RUNX2(cc+/#2) and /#3 were more mislocalized and aggregated in cells than wt RUNX2 (*P* < 0.001 versus wt, Tukey HSD *post hoc* test, in all instances). Indeed, RUNX2(cc+/#2) and /#3 did not differ from RUNX2(+12A), both in terms of distribution and aggregation (*P* > 0.65 versus RUNX2(+12A), Tukey HSD *post hoc* test, in all instances; Fig. 4E and G). These findings show how the effects of CC stabilization in the polyQ/polyA region of RUNX2 on protein localization/aggregation closely mimic those of polyA expansion, supporting the notion that polyA expansion triggers protein aggregation *in vivo* through CC stabilization. Furthermore, these results indicate a more prominent role of the polyA CC segment, with respect to the polyQ section, in the regulation of RUNX2 localization/aggregation. Interestingly, we also found through co-expression experiments that non-expanded wt RUNX2 is recruited into aggregates formed either by polyA-expanded RUNX2(+12A) or by the CC-stabilized RUNX2(cc+/#2) mutant (Fig. 5A). These findings indicate that the excessive elongation/stabilization of the RUNX2 polyQ/polyA CC domain induces not only protein aggregation but also sequestration of wt RUNX2, which may obviously play a role in the molecular pathogenesis of cleidocranial dysplasia.

Next, we tested the effect on the localization/aggregation of polyA-expanded RUNX2 of CC-disrupting mutations in the polyQ stretch, in the polyA stretch, or in both. We found that polyQ CC-disruption [mutant RUNX2(+12A/cc−/#1), Fig. 4D, left panels] moderately, but significantly, reduces mislocalization and aggregation with respect to RUNX2(+12A) [*P* < 0.01 versus RUNX2(+12A) for aggregation, *P* < 0.04 for mislocalization, Tukey HSD *post hoc* test; Fig. 4F and H]. CC disruption in the expanded polyA stretch [mutants RUNX2(+12A/cc−/#2) and /#3; Fig. 4D, middle and right panels] strongly reduces both mislocalization and aggregation with respect to the polyA-expanded form of RUNX2 [*P* < 0.001 versus RUNX2(+12A), Tukey HSD *post hoc* test, in all instances; Fig. 4F and H]. These results somewhat mirror those obtained with the *cc+* mutants in showing how the stability of the expanded polyQ/polyA CC of RUNX2 critically regulates the localization and aggregation of the mutant protein, highlighting again a more prominent role of the polyA with respect to the polyQ segment in RUNX2.

Finally, the aggregation propensity in cells of wt and mutant forms of RUNX2 was also assayed at the biochemical level, by analyzing with dot immunoblotting the distribution of the different RUNX2 forms in supernatant and pellet fractions of cell lysates after ultracentrifugation (Fig. 5B and C), by which protein aggregates tend to be enriched in the pellet fraction, whereas soluble proteins tend to remain in the supernatant. We had previously used a similar approach to characterize CC mutants of huntingtin (15). The results of this experiment closely parallel what we observed with confocal microscopy. In fact the aggregation-prone, CC-stabilized mutant RUNX2(cc+/#3) was enriched in the pellet fraction similar to the polyA-expanded RUNX2(+12A) mutant. Conversely, the CC-destabilized mutant RUNX2(+12A/cc−/#3) had a distribution comparable with that of wt RUNX2. One-way ANOVA [*F*_(3,27)_ = 8.36, *P* < 0.001, *n* = 7–8 in each group] revealed significant differences between RUNX2 and both RUNX(+12A) and RUNX2(cc+/#3) (*P* < 0.03 and *P* < 0.01, respectively, Tukey HSD *post hoc* test), and between RUNX2(+12A) and RUNX2(+12A/cc−/#3) (*P* < 0.03). Together with the confocal microscopy analyses of aggregation, this experiment further supports the notion that the stability of the polyQ/polyA CC is a key determinant of RUNX2 aggregation.

### CC length and stability control aggregation of polyA and polyQ/polyA CC fragments *in vivo*

**Figure 6.**
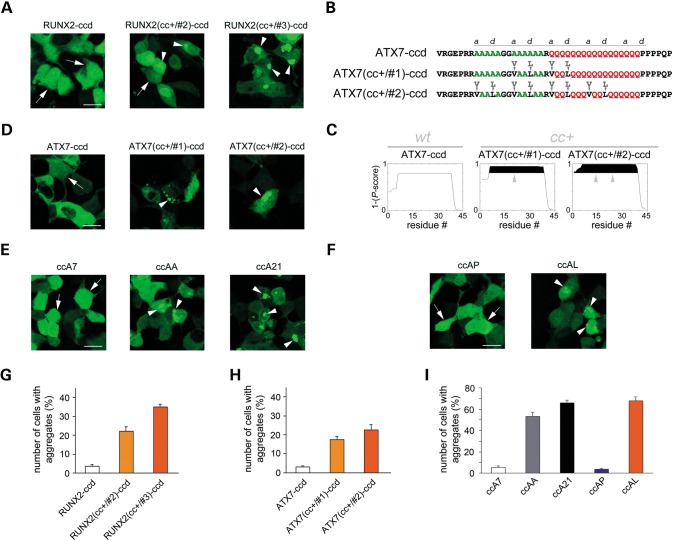
Aggregation of polyQ/polyA CC domains and polyA peptides. (**A**) Confocal fluorescence images of cells expressing the GFP-tagged CC domain of RUNX2 (RUNX2-ccd, left panel), or CC-stabilized variants of it (middle and right panels). Arrowheads indicate intracellular aggregates. Arrows indicate cells with diffuse fluorescence. Calibration bar: 20 µm. (**B**) Primary sequence of the polyA/polyQ CC domain of ATX7 with a few flanking residues (ATX7-ccd), and CC-stabilized variants of it, i.e. ATX7(cc+/#1)-ccd and ATX7(cc+/#2)-ccd. Substitutions with CC-stabilizing amino acids are highlighted (arrowheads and letters) above each sequence. (**C**) CC propensity of ATX7-ccd variants shown in B as determined by Paircoil2, expressed as 1 minus the *P*-score assigned to each amino acid in the primary sequence. Protein segments whose CC propensity is 0.8–1 are highlighted in black. Vertical arrowheads indicate the site and effect of CC-stabilizing mutations. (**D**) Confocal fluorescence images of cells expressing the GFP-tagged CC domain of ATX7 (ATX7-ccd, left panel) or CC-stabilized variants of it (middle and right panels). Labeling and calibration as in A. (**E**) Confocal fluorescence images of cells expressing the GFP-tagged polyA peptides ccA7, ccAA and ccA21, which contain at their core a stretch of 7, 14 and 21 alanines, respectively. Labeling and calibration as in A. (**F**) Confocal fluorescence images of cells expressing GFP-tagged variants of peptide ccAA bearing CC-destabilizing (peptide ccAP) or CC-stabilizing (peptide ccAL) mutations. Labeling and calibration as in A. (**G**) Percentage of cells overexpressing for 48 h GFP-tagged wt or CC-stabilized forms of RUNX2-ccd that contain aggregates. (**H**) Percentage of cells overexpressing for 48 h GFP-tagged wt or CC-stabilized forms of ATX7-ccd that contain aggregates. (**I**) Percentage of cells overexpressing for 48 h GFP-tagged polyA CC peptides that contain aggregates.

To further confirm that polyQ/polyA CCs can trigger the aggregation process, we tested whether just the isolated CC fragments of aggregation-prone RUNX2 mutants can aggregate when expressed in cells. To this aim, we generated expression plasmids encoding for the GFP-tagged CC domain (ccd) of RUNX2 with a few flanking residues (RUNX2-ccd), encompassing exactly the same tract of the RUNX2 protein the we studied with CD (peptide RUNX2-ccd), or for mutant forms of the same domain with CC-stabilizing mutations such as those present in RUNX2(cc+/#2) and RUNX2(cc+/#3), i.e. peptides RUNX2(cc+/#2)-ccd and RUNX2(cc+/#3)-ccd. RUNX2(cc+/#3)-ccd has the same sequence as the peptide RUNX2(cc+)-ccd that was studied with CD experiments. When overexpressed in HEK293 cells, these peptides recapitulated the behavior of full-length RUNX2 and *cc*+ mutants (Fig. 6A and G). In fact, both RUNX2(cc+/#2)-ccd and RUNX2(cc+/#3)-ccd formed numerous, often large, aggregates in 21–35% of the cells, respectively, whereas RUNX2-ccd had an essentially diffuse distribution, occasionally forming small aggregates only in few overexpressing cells (*F*_(2,33)_ = 65.29, *P* < 0.001, one-way ANOVA; *P* < 0.001, Tukey HSD *post hoc* test for RUNX2-ccd versus either RUNX2(cc+/#2)-ccd or RUNX2(cc+/#3)-ccd; *n* = 12 microscopic fields in all instances) (Fig. 6G). This conclusion was also corroborated by an analogous experiment with the polyA/polyQ CC domain of ATX7, a polyA/polyQ protein mediating spinocerebellar ataxia 7 upon polyQ expansion (5). We generated constructs for the *in vivo* expression of the CC domain of a non-expanded form of ATX7 [i.e. ATX7-ccd (Fig. 6B), comprising two short stretches of polyA and a stretch of 17 Qs], and of two different CC-stabilized forms of it [i.e. ATX7(cc+/#1)-ccd and ATX7(cc+/#2)-ccd] with an increasing number of CC-stabilizing mutations, as shown in Figure 6C. In fact, one-way ANOVA [*F* =_(2,45)_ = 25.65, *P* < 0.001] showed significant differences in the aggregation rate between ATX7-ccd and both the ATX7(cc+/#1)-ccd and ATX7(cc+/#2)-ccd mutants (Fig. 6D and H; *P* < 0.001, Tukey HSD *post hoc* test for ATX7-ccd versus both mutants, *n* = 16 fields in all instances). Strikingly, the same phenomena were observed even when we overexpressed in HEK293 cells the polyA CC model peptides that we had studied *in vitro*. GFP-tagged peptides ccAA and ccA21, which form CC structures and polymers *in vitro*, readily aggregated in the cellular environment in a polyA length-dependent manner (*F*_(4,35)_ = 138.9, *P* < 0.001, one-way ANOVA) (Fig. 6E). In fact, a similar peptide with only seven alanines (ccA7) formed aggregates only in a small number of overexpressing cells, whereas ccAA and ccA21 formed aggregates in more than half of the overexpressing cells (*P* < 0.001 for ccA7 versus both ccAA and ccA21, Tukey HSD *post hoc* test) (Fig. 6I). Furthermore, CC-stabilizing (peptide ccAL) and -destabilizing mutations (peptide ccAP) in the polyA stretch, respectively, enhance or abolish aggregation, similarly to what was observed with similar mutations in full-length and ccd RUNX2 (Fig. 6F). Notably, the aggregation rate of the ccAA peptide (53.4 ± 3.9%, *n* = 8 fields) is enhanced by either elongation of the polyA tract (peptide ccA21, 66.0 ± 2.4%, *n* = 8, *P* < 0.02, Tukey HSD *post hoc* test) or by its stabilization (peptide ccAL, 68.0 ± 3.4%, *n* = 8, *P* < 0.01, Tukey HSD *post hoc* test) (Fig. 6I). Taken together, these findings indicate that—in two different disease-related proteins as well as in model peptides—the stability/length of polyQ/polyA and polyA CCs is a key determinant of the aggregation proneness, thus indicating the generality of the CC-triggered polyQ and polyA aggregation mechanism.

### Modulation of RUNX2 activity by length and stability of its polyQ/polyA CC

**Figure 7.**
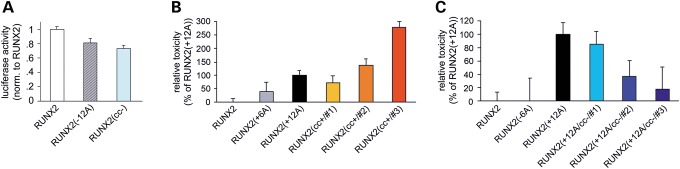
Transcriptional activity and cellular toxicity of wt and mutant forms of RUNX2. **(A**) Relative firefly luciferase activity in lysates of HEK293 cells co-expressing firefly luciferase under the 6xOSE2 promoter, Renilla luciferase under the CMV promoter and the indicated RUNX2 constructs, as measured 48–72 h after transfection. For each sample, values of firefly luciferase luminescence were normalized to those of Renilla luciferase. All values were then normalized to those of wt RUNX2. (**B**) Relative cellular toxicity of GFP-tagged wt, polyA-expanded and *cc+* mutant RUNX2 after 72 h of overexpression. Data are normalized to RUNX2(+12A). (**C**) Relative cellular toxicity of GFP-tagged wt, polyA-expanded, partially polyA-deleted or *cc−* mutant RUNX2 after 72 h of overexpression. Data are normalized to RUNX2(+12A).

We also analyzed whether the length and stability of the polyQ/polyA CC also regulates the physiological transcriptional activity of RUNX2. Evidence exists that either partial deletions of the polyA stretch or short expansions of the polyQ stretch determine a decreased transcriptional activity of RUNX2 (43). To determine the relevance of the CC structure of the polyQ/polyA repeats to the physiological function of RUNX2, we have investigated whether—besides the length of the polyA stretch—also the stability of its CC structure has relevance to the transcriptional activity of RUNX2. To this aim, we have used a luciferase reporter assay to compare the transcriptional activity of wt RUNX2 with that of a polyA deletion mutant [i.e. RUNX2(−12A)] and of a mutant in which the stability of the non-expanded polyA CC structure is disrupted by alanine-to-proline mutations, i.e. RUNX2(cc−) (Fig. 3A and B). This experiment revealed that CC disruption in the polyA stretch of RUNX2 negatively modulates its transcriptional activity, paralleling the effect of polyA deletion [*F* = _(2,61)_ = 8.25, *P* < 0.01, one-way ANOVA] (Fig. 7A). In fact, the transcriptional activity of RUNX2(cc−) was 0.73 ± 0.04 times that of wt RUNX2 (*P* < 0.01, Tukey HSD *post hoc* test) Similarly, the RUNX2(−12A) deletion mutant had a reduced level of activity (*P* < 0.03, Tukey HSD *post hoc* test versus wt RUNX2). These findings indicate that, besides the overall length, also the structure of the polyQ/polyA CC domain of RUNX2 is relevant to its physiological function.

### Increased length and stability of the RUNX2 polyQ/polyA CC induce cellular toxicity

PolyA-expansion in genetic diseases is associated not only with protein aggregation/dysfunction but also with cellular toxicity (3). To assess the role of CCs in the toxicity of polyA-expanded RUNX2, we overexpressed in HEK293 cells GFP-tagged RUNX2, or RUNX2(+12A), or their *cc+* and *cc−* mutants, and used a colorimetric MTT-formazan assay to determine their relative toxicity (44) (Fig. 7B and C).

Overall, one-way ANOVA revealed significant differences among groups related to the overexpression of the different RUNX2 mutants [*F*(9,462) = 8.63, *P* < 0.001]. We observed some significant toxicity after 72 h of RUNX2(+12A) overexpression with respect to wt RUNX2 used as a control (i.e. formazan production was, respectively, 89.3 ± 1.8% in *n* = 94 culture wells *versus* 100.0 ± 1.4% in *n* = 119 wells, *P* < 0.01, Tukey HSD *post hoc* test; values normalized to wt). Conversely, more limited expansions or partial deletions of the polyA stretch in RUNX2(+6A) and RUNX2(−6A) did not cause evident toxicity (*n* = 39 and *n* = 36, respectively, *P* > 0.90 versus wt in both cases). To compare the relative toxicity of the CC mutants, we normalized the toxicity of each mutant to that of polyA-expanded RUNX2(+12A) (Fig. 7B). Strikingly, the RUNX2(cc+/#2) in which the polyA CC was stabilized was as toxic as the polyA-expanded form RUNX2(+12A) (*n* = 76, *P* < 0.001 versus wt, Tukey HSD *post hoc* test). The RUNX2(cc+/#1) mutant, in which the polyQ part of the CC was stabilized, showed only a modest, non-significant degree of toxicity. However, when both the polyQ and polyA sections of the CC were stabilized as in RUNX2(cc+/#3), the toxicity was even higher than that of RUNX2(cc+/#2), more than 2-fold as compared with RUNX2(+12A) (*n* = 23, *P* < 0.001 versus wt, Tukey HSD *post hoc* test), indicating that the overall stability of the polyQ/polyA CC is relevant to toxicity.

Mirroring these results, polyA-expanded mutants with CC-destabilizing mutations (Fig. 7C) showed instead a very marked loss of toxicity when the CC-destabilizing residues were positioned within the polyA stretch [i.e. mutant RUNX2(+12A/cc−/#2) and /#3; *P* = 0.95 versus wt and *P* = 0.99 versus wt, respectively, Tukey HSD *post hoc* test], whereas the destabilization of the RUNX2(+12A) polyQ portion by itself reduced toxicity partially but not significantly [mutant RUNX2(+12A/cc−/#1)].

Taken together, these findings indicate a critical role of the overall length and stability of the polyQ/polyA CC of RUNX2 for the induction of cellular toxicity. The greater relevance to toxicity of the polyA section of the RUNX2 CC with respect to the polyQ section is also evident, paralleling what was observed for subcellular distribution and aggregation.

## DISCUSSION

The results of our study identify in numerous proteins of human and non-human eukaryotic proteomes a systematic association of polyQ, polyA and polyQA repeats with each other, and with CC domains, of which they can be a part. These associations appear to be dynamically evolving throughout phylogenesis and have implications for human genetic diseases in which polyQ or polyA repeats, associated or not, are pathologically expanded.

### Philogenetically graded occurrence and association of polyQ and polyA repeats

PolyQ and polyA repeats are widespread in proteomes (12). Their co-occurrence in the same proteins has also been occasionally noticed in different proteomes (11,10), but the general significance of their association is not clear. PolyQA repeats have also been observed in some proteins (7,8), but little is known about their general association with polyQ and polyA repeats.

We have systematically screened six complete eukaryotic proteomes for the occurrence and co-occurrence in proteins of polyQ, polyA and polyQA repeats of four or more residues. This length threshold allowed us to detect both long repeats (e.g. RUNX2), as well as cryptic low-complexity regions (45) composed of short, often intermixed stretches of these amino acids (e.g. PQN-41) that would go unnoticed using higher thresholds. Our analyses reveal a novel phylogenetically graded association between polyQ, polyA and polyQA repeats in the proteins of human and non-human eukaryotic proteomes, raising new questions regarding the origin and the possible functional significance of this association.

We have found that the absolute percent occurrence of polyQ and polyA proteins in different species is quite variable, as previously observed in the literature (18,46), with a weak correlation with phylogenetic distances. Amino acid repeats in proteins originate by DNA triplet repeat expansions caused by replication slippage and/or unequal crossing-over (47,48). Species-specific differences in the frequency of these phenomena may contribute to the differences in the overall occurrence of repeats in different proteomes that we and others have observed (49,50). However, we also observed that the ratio between the percentage of polyA and polyQ proteins varies with regularity throughout phylogenesis in the proteome of six species ranging from yeast to humans, correlating strongly with phylogenetic distances, despite the observed differences in the absolute occurrence of polyA and polyQ proteins in the different species. This indicates that the regular variation of the polyA/polyQ protein ratio is not just the result of two opposite trends in the absolute occurrence of polyQ and polyA repeats in proteomes throughout phylogenesis but is an index of related evolutionary dynamics in the occurrence of these two repeats. This view is supported by two other observations. First, the polyQ and polyA protein groups overlap significantly more than expected by chance (Q4 + A4 proteins) in all the proteomes that we analyzed, and, second, the overlap between these two groups when normalized to the percentage of polyA proteins in each proteome varies also with regularity throughout phylogenesis, i.e. proteomes with more polyA proteins tend to have a higher proportion of these containing also a polyQ stretch, and this phenomenon gradually attenuates throughout phylogenesis.

A combination of different genetic and evolutionary mechanisms may have shaped these phylogenetic dynamics in the occurrence and co-occurrence of polyQ and polyA repeats, including GC pressure (51), positive and negative selection (1,46,52,53) and frameshift mutations (54) that stably encode in the genome polyQ-to-polyA frameshifts such as those occurring at the transcriptional or translational level (55,56,57). These and other conceivable mechanisms that may underlie the evolutionary dynamics and associations of polyQ, polyA and polyQA repeats are not mutually exclusive. We have indeed evidence that they actually concur and are also acting for associations of other amino acid repeats throughout phylogenesis (F. Fiumara *et al*., in preparation). Phylogenetic dynamics similar to those that we observed for polyQ and polyA repeats characterize also polyQA repeats. PolyQA repeats can originate from expansion of QA- or AQ-encoding hexanucleotides originated after point mutations within polyQ- or polyA-encoding triplet repeats (58), and this may promote their co-occurrence with both polyA and polyQ repeats.

### CC structural propensity of polyA and polyQ repeats

Our experiments show that, besides being significantly associated in eukaryotic proteomes, polyQ and polyA repeat share a common structural propensity to form α-helical CC structures. We had previously found that polyQ repeats have CC propensity and are frequently found within conventional CC segments (15). Here we find that polyA repeats as well have a significant association with CC domains in human and non-human proteomes and have propensity to form these α-helical super-secondary structures. In fact, our analyses showed that polyQ, polyA and mixed peptides, such as those encompassing the polyQ/polyA domain of RUNX2, display typical CD signatures of CC structures. These results are consistent with the well-known α-helical propensity of polyA stretches (23,24,59). In a polyA CC, alanines occupy all heptad positions, including the *a/d* core, and this is also consistent with previous observations on *alacoils*, alanine-zippers and other alanine-rich sequences, i.e. CC structures containing heptad-spaced alanines at core positions in conventional non-polyA CC heptad repeats (60,61,62,63), and with studies showing how conventional CCs can tolerate the insertion of short polyA sequences (29).

PolyA stretches can interact with each other and form soluble multimers in a length-dependent manner (24,64). Our CD and chemical cross-linking experiments show how CC structures can mediate both the self-interaction and multimerization of polyA sequences. This CC-mediated multimerization is related to the stability of the polyA CC. The stability of such structure is increased by polyA elongation, which mimics the effects of stabilization induced by hydrophobic residues in *a/d* positions. Interestingly, the ccA21 peptide, which contains a polyA stretch within the range of those causing human disease (3) and displays marked polymerization tendency *in vitro*, forms hierarchically organized fibrillar structures that intertwine in complex tangles, as shown by AFM analyses. These structures, which bear resemblance to those formed by other CCs (38) and polyQ proteins (37) may well represent the substrate of aggregation *in vivo*.

We have also found that polyA repeats can often associate with polyQ and polyQA repeats in the context of CC-prone protein domains. To determine the possible structural/functional significance of these associations, we studied CC peptides containing pure repeats as well as repeat combinations and found that polyA CCs are relatively more stable than polyQ CCs, and that mixed polyA/polyQ or /polyQA peptides have intermediate degrees of stability. A sequence/structure combinatorial code could finely tune the relative stability of complex polyQ, polyA and polyQA CCs such as those actually found in proteomes, providing a possible explanation as to why this type of sequences is maintained, and subtly varied, throughout phylogenesis. For instance, the polyQ/polyA length polymorphism and the polyQ/polyA length ratio in RUNX2 regulates its transcriptional activity and craniofacial variation in dogs and other carnivores (65,66), and RUNX2 polyQ/polyA length polymorphism is related to differences in bone mineral density in humans (43). Our findings indicate that these skeletal variations may be related to graded variations in the stability of the RUNX2 polyQ/polyA CC caused by the observed polymorphisms in the relative length of these repeats. Moreover, the results of our luciferase assay experiments also support this view by indicating that, besides the length, also the stability of the polyQ/polyA CC has a role in modulating the physiological transcriptional activity of RUNX2.

Furthermore, our observations have obvious relevance to human skeletal diseases such as cleidocranial dysplasia in which the polyQ/polyA CC structure of RUNX2 is extended by polyA expansion.

### Coiled-coil stability as a determinant of polyA-expanded protein aggregation and toxicity

PolyQ- and polyA-expansion diseases are related to the excessive elongation of polyQ or polyA repeats beyond a critical threshold. These expansions lead to protein aggregation and dysfunction/toxicity, which are variably related to gain- or loss-of-function phenomena, causing severe developmental or neurodegenerative diseases (2,3). We have used the polyQ/polyA transcription factor RUNX2 as a model for studying *in vivo* the role of CC structures in pathological protein aggregation and dysfunction upon polyA expansion, as occurring in cleidocranial dysplasia. By structure-guided mutagenesis, we have generated wt and polyA-expanded variants of RUNX2 with enhanced or reduced CC stability and found that the stability of the RUNX2 CC has a critical role in determining the subcellular localization, aggregation and toxicity of the protein.

Consistent with previous reports (41,42), wt RUNX2 had an essentially nuclear localization, whereas polyA-expanded RUNX2 was often prevalently cytoplasmic, forming multiple aggregates. We found that mutants in which the polyA CC was stabilized by hydrophobic residues (*cc+*) displayed distribution and aggregation patterns comparable with polyA-expanded RUNX2. Conversely, when the polyA-expanded CC was destabilized (*cc−*), mislocalization and aggregation were substantially reduced. These findings closely parallel what was observed in CD and cross-linking experiments with model peptides in which polyA CC structures were stabilized and made more prone to multimerize, either by polyA elongation (peptide ccA21 versus ccAA) or by substitutions with CC stabilizers (ccAL versus ccAA), but were instead disrupted by CC-destabilizing mutations (ccAP versus ccAA). Even more notably, there was a clear correspondence between the differential CC stability of the RUNX2-ccd and RUNX2(cc+)-ccd peptides and their respective *in vitro* polymerization and *in vivo* aggregation behavior. In fact, CC-stabilized RUNX2(cc+)-ccd displayed a greater thermal stability as compared with its wt counterpart, formed higher-order multimers/polymers *in vitro* and had a marked aggregation tendency when expressed in living cells [i.e. RUNX2(cc+/#3)-ccd], mimicking the aggregation behavior of full-length RUNX2(cc+/#3). Similar phenomena were observed by overexpressing the CC domain of ATX7, i.e. the aggregation of non-polyQ-expanded ATX7-ccd can be triggered by CC-stabilizing mutations, thus showing the generality of these CC-triggered aggregation mechanisms. Furthermore, even model polyA peptides were able to form *in vivo* aggregates in a polyA length-dependent manner. In particular, the aggregation rate of a peptide with 14 alanines (peptide ccAA) was enhanced by either CC-stabilizing mutations (peptide ccAL) or by polyA elongation to 21 residues (peptide ccA21). Again, the *in vivo* aggregation behavior of ccAA, ccA21 and ccAL correlated strongly with their respective CC stability as revealed by CD. Thus, based on the biophysical and cell biological experiments that we performed, polyA expansions can be seen as inducing a critical increase in CC stability that promotes excessive multimerization and ultimately pathological aggregation.

Besides polymerization and aggregation, CC structures can mediate also homotypic and heterotypic protein–protein interactions (16). The mislocalization of the protein may result from aberrant protein–protein interactions mediated by pathologically elongated CCs (15), retaining the protein in the cytoplasm after synthesis and/or altering its physiological nucleo-cytoplasmic shuttling (67). Abnormal CC interactions may also cause entrapment of other proteins into aggregates causing protein-loss-of-function and toxicity through this mechanism (15). Cleidocranial dysplasia associated with polyA expansion in RUNX2 is, like other polyA and polyQ diseases, a dominant genetic disease, i.e. in heterozygosis, cells contain also non-polyA-expanded copies of RUNX2 produced from the normal allele. Our experiments show that aggregates formed by polyA-expanded—or otherwise CC-stabilized—RUNX2 can sequester into cytoplasmic inclusions even wt RUNX2. The loss-of-function of RUNX2, associated with polyA-expansion or with other mutations, is a fundamental pathogenetic mechanism of cleidocranial dysplasia (39). The observed entrapment of wt RUNX2 into the aggregates can induce RUNX2 loss-of-function by subtracting to the nuclear compartment even the normal copies of the protein that are present in the cell.

The composite nature of the RUNX2 polyQ/polyA CC allowed us to define the relative contribution of the polyQ and polyA sections to protein localization and aggregation. CC-stabilizing and -destabilizing mutations restricted to the polyQ stretch induced much weaker phenotypes than those determined by the same mutations in the polyA stretch. Together with the *in vitro* experiments, these observations indicate that polyA CCs have a greater stability as compared with polyQ CCs of the same length. There is evidence for the evolutionary selection of shorter and less polymorphic polyA repeats with respect to polyQ repeats (68). At the same time, the disease threshold for polyA expansions is lower than that for polyQ expansions (2,69). The fact that polyA CCs are more stable than polyQ CCs can rationalize both phenomena in structural terms, i.e. polyA CCs become aggregation-prone and harmful to cells when they reach comparatively shorter lengths than polyQ CCs and are therefore under tighter evolutionary constraints.

We have also studied the role of CC structures in the toxicity of polyA expansions. We have found that polyA-expanded RUNX2 has some toxicity when overexpressed in cells, similar to what was observed with other polyA-expanded proteins (3), and that *cc+* mutants of non-expanded RUNX2 are as toxic, or even more toxic, than polyA-expanded RUNX2. Moreover, the toxicity related to the polyA expansion could be significantly reduced or abolished by destabilizing the CC (*cc−* mutants). Again, stabilization of the polyA section of the CC had a substantially greater effect than that of the polyQ section, in good agreement with previous studies comparing the relative toxicity of polyQ and polyA expansions in Ataxin-7 (70). The toxicity of polyQ- and polyA-expanded proteins is thought to rely on several mechanisms, including sequestration of the wt non-expanded protein and other proteins into aggregates, as well as aberrant protein–protein interactions. Our findings suggest that, as for polyQ expansions (15), polyA expansions may trigger these mechanisms through non-physiological CC-mediated interactions of the mutant protein that lead to toxic gain-of-function effects. Interestingly, while the most CC-stabilized RUNX2(cc+/#2) and (cc+/#3) mutants induced aggregation at levels comparable with those of the polyA-expanded mutant RUNX2(+12A), their toxicity level could be even two-three times higher than that of RUNX2(+12A). Aggregation and toxicity in polyQ and polyA-expansion diseases do not necessarily go in parallel. In fact, aggregates may not represent the only toxic entities in these diseases (71–75) and may even have protective roles (76,77), although they can impair several cellular functions (78,79). Rather, dysfunctional toxic oligomers/multimers are thought to have a fundamental role in the molecular pathogenesis of these disorders (80). Our experiments strongly suggest that CC-based oligomers/multimers may represent the fundamental determinants of protein dysfunction and toxicity in polyA-expansion diseases such as cleidocranial dysplasia.

The molecular pathogenesis of polyA disease is generally thought to rely on protein misfolding events by which expanded polyA stretches would assume a β-sheet-rich conformation, closely paralleling the behavior of other well-studied conventional amyloids such as Aβ(1–42) (3,81). The structure of polyA sequences has been extensively used as a helical model system by both molecular modeling approaches and structural studies on peptides (24,82–85). Besides the α-helical structure (23,24,83), some polyA-based peptides have been shown to be able to assume *in vitro* multiple conformations, including β-sheets (86,87) and PP-II structures (88). This fact has suggested that misfolding to aggregation-prone β-sheets could represent the essential mechanism of polyA-expansion diseases, causing protein aggregation and toxicity in analogy with other known amyloid diseases (14). However, as noted by Bernacki and Murphy (24), in those studies in which the conversion to β-sheets was observed, the peptides were under substantially non-physiological conditions of temperature, pH and concentration (86,87). Studies in benign buffers under more physiological conditions, similar to those we employed, consistently observed α-helical secondary structures even in very long polyA stretches and did not show conversion to β-sheets (23,24,83). Rather, a critical observation in these and other studies is that the expansion of polyA stretches leads to an increase in helical structure (24,59,89). Our experiments extend these observations and show that polyA expansion is related to a substantial stabilization of the CC super-secondary structure, with a parallel increase in multimerization propensity. These lines of evidence support the notion that the assembly of CC structures rather than β-sheets primarily triggers the aggregation and toxicity of polyA-expanded proteins, as previously observed for polyQ proteins (15). This CC model does not exclude at all the possibility that CCs are intermediate structures in the aggregation process, and polyA β-sheets are subsequently formed at the level of multimers or aggregates/fibers (15,90,91). Furthermore, our findings are also compatible with the notion that polyA stretches may promote aggregation and/or toxicity in association with other protein domains (15,92,93).

In this view, the expansion of polyA and polyQ stretches leads primarily, rather than to misfolding, to an excessive enhancement of their native fold, which may ultimately explain the pathological enhancement of their native functions, an emerging gain-of-function mechanism observed in triplet-expansion diseases (94–96), as well as the triggering of protein aggregation and mislocalization causing protein loss-of-function (2,3). In conclusion, this work identifies a novel phylogenetic and structural association between polyQ, polyA and polyQA repeats, which has implications for both the physiological function of the proteins in which these repeats are normally found, and for the molecular pathogenesis of polyQ- and polyA-expansion diseases, providing for these a unified structural framework in which CC assembly and dynamics play a critical role.

## MATERIALS AND METHODS

### Bioinformatics

The FASTA protein sequences of the complete reference proteomes of *Homo sapiens*, *Mus musculus*, *Xenopus tropicalis*, *Drosophila melanogaster*, *Caenorhabditis elegans* and *Saccharomyces cerevisiae* were downloaded from the Uniprot online database of complete reference proteomes (available at http://www.uniprot.org). Manually reviewed entries were considered for *Homo*, *Mus*, and *Saccharomyces* proteomes, and all entries were considered for the other three species whose manual review process is at early stages (see http://www.uniprot.org). The Paircoil2 CC-prediction software (27) was downloaded from http://groups.csail.mit.edu/cb/paircoil2/. *Ad hoc* Perl scripts (http://www.perl.org) were generated to identify, in each proteome, the proteins containing stretches of glutamine (Q), alanines (A) or glutamine-alanine (QA) of at least four residues, as well as proteins containing predicted CC domains according to Paircoil2, using a *P*-score <0.05 as a detection threshold. Finally, the overlap between predicted CC segments and Q, A and QA repeats was measured for each protein, and the number of proteins with an observed overlap of <4 or ≥4 residues was determined. The overlap expected by chance was calculated considering the relative percent lengths of the CC domain(s) and of the repeat region(s) in each protein, and the number of proteins with an expected overlap of <4 or ≥4 residues was then determined. Observed and expected values were then compared statistically (see the section Results). Data were analyzed quantitatively using Excel (Microsoft). Phylogenetic distances between the six species were estimated based on the time of evolutionary divergence of each species from *Homo sapiens* as derived from http://www.timetree.org (26). Venn diagrams were generated using the BioVenn software (97).

### Plasmids

The RUNX2 DNA sequence was PCR-amplified using a proof-reading polymerase (Agilent) from a RUNX2-encoding plasmid (clone HsCD00295185; obtained from the PlasmId repository, Harvard University) and cloned into a pENTR/D entry vector (Invitrogen). The RUNX2 sequence was then subcloned in frame with Em-GFP into the pcDNA6.2/C-EmGFP-DEST destination vector (Invitrogen) by LR recombination (Gateway system, Invitrogen), to generate an expression vector for the RUNX2-EmGFP fusion protein. For simplicity, we refer to EmGFP as GFP. The RUNX2 sequence was also subcloned into another destination vector (pcDNA6.2/V5-DEST) in frame with a V5 tag by LR recombination. Wild-type and mutant forms of RUNX2 were also PCR-amplified and cloned into the pDsRed-Monomer-N In-Fusion Ready plasmid (Clontech) using the In-fusion cloning system (Clontech), following the manufacturer's protocol, to generate DsRed-tagged constructs. The constructs encoding for GFP-tagged ccd fragments of RUNX2 and ATX7, and for GFP-tagged CC model peptides (ccA7, ccAA, ccA21, ccAP and ccAL) were generated by cloning of PCR products, amplified using Taq polymerase (Sigma), into the pcDNA3.1/CT-GFP expression vector (Invitrogen), following the manufacturer's procedure. The PCR products encoding for RUNX2-ccd, RUNX2(cc+/#2)-ccd and RUNX2(cc+/#3)-ccd were generated using as a template the pcDNA6.2/C-RUNX2-GFP, pcDNA6.2/C-RUNX2(cc+/#2)-GFP and pcDNA6.2/C-RUNX2(cc+/#3)-GFP expression vectors, respectively. The PCR products encoding for ATX7-ccd, ATX7(cc+/#1)-ccd, ATX7(cc+/#2)-ccd, ccA7, ccAA, ccA21, ccAP and ccAL were generated from partially overlapping synthetic DNA primers (Sigma). In all cloning procedures, PCR products were gel-purified using the QIAquick Gel Extraction kit (Qiagen). Plasmids were amplified and purified using the QIAprep Spin Miniprep kit and the HiSpeed Plasmid Maxi kit (Qiagen).

### Peptides

The synthetic peptides used in this study were obtained from Genway Biotech (San Diego, California, USA) with >95% purity, N-terminal acetylation and C-terminal amidation. Lyophilized peptides were dissolved in a benign buffer (100 mm NaCl and 10 mm phosphate buffer, pH 7.4) to obtain concentrated stock solutions (2–5 mg/ml) that were used immediately for CD experiments and then aliquoted and stored at −80°C for subsequent experiments.

### Circular dichroism spectropolarimetry

The structure of peptides was studied trough CD using a J-815 spectropolarimeter (Jasco). Peptides were dissolved in 100 mm NaCl and 10 mm phosphate buffer, pH 7.4 at a concentration of 0.1–0.3 mg/ml, and spectra (190–260 nm) were acquired at the desired temperatures measuring the ellipticity of peptide samples in 0.1-mm quartz cuvettes every 0.5–1 nm (from 195 to 260 nm). The mean residue molar ellipticity ([θ]) was calculated from the formula [θ] = θ × mw/10 × (*n*–1) × c × pl, where *θ* is the measured ellipticity, mw is the molecular weight of the peptide, *n* is the number of amino acids in the peptide, c is the concentration of the peptide (mg/ml), and pl is the cuvette pathlength (cm) (36). To test the thermal stability of peptides, the samples were heated from 5 to 75°C (10°C/min) while recording ellipticity at 222 nm. Circular dichroism data and graphs were elaborated and smoothed using Spectra Manager (Jasco) and Excel (Microsoft) software.

### Chemical cross-linking

The oligomeric state of peptides was studied using chemical cross-linking with glutaraldehyde, as previously described (15). Briefly, peptides stocks (diluted to 0.6–1.2 mg/ml in benign buffer) were incubated for 15 min with 0.01% glutaraldehyde (Sigma) at 37°C, or with 0.05% glutaraldehyde at 15°C. These two conditions were alternatively chosen to better highlight subtle differences between members of the different peptide sets that were analyzed. The reaction was stopped by adding ethanolamine (Sigma) to a final concentration of 0.1 m. Cross-linked and non-cross-linked peptides (usually ∼2 µg and ∼4–5 µg for RUNX2-ccd and RUNX2(cc+)-ccd peptides, as these two peptides were less efficiently silver-stained than the other peptides) were electrophoretically separated by Tricine–SDS–PAGE (98) and silver-stained using the SilverQuest kit (Invitrogen) following the manufacturer's procedure. The electrophoretic mobility of peptides containing long polyQ stretches was slightly reduced with respect to what was expected based on their molecular weight, as previously observed for other polyQ-containing proteins.

### Atomic force microscopy

AFM images were collected under ambient conditions in tapping mode by means of an Easyscan2 AFM (Nanosurf) equipped with a 10-µm scan head. Peptide stock solutions were incubated at 4°C for at least 72 h, a time frame during which their CD spectra retained their α-helical CC features. Stock solutions before analysis were diluted to 0.05–0.25 mg/ml in benign buffer and dropped onto freshly cleaved mica (Ted Pella, Inc.) for 1–2 min. The mica surface was then gently washed with ultrapure water and dried under a moderate N_2_ stream.

### Site-directed mutagenesis

Site-directed mutagenesis of RUNX2-encoding plasmids was performed using the QuikChange Lightning Multi Site-Directed Mutagenesis Kit (Agilent), following the manufacturer's protocol. Mutagenic primers were designed using the QuikChange Primer Design program, available at http://www.genomics.agilent.com. The presence of the desired mutations was verified through DNA sequencing.

### Cell culture, transfection and imaging

HEK293 cells (293F strain; Gibco-Invitrogen) were maintained following standard procedures in Dulbecco's modified Eagle's medium supplemented with 10% fetal bovine serum. According to the experimental requirements, the cells were plated either in 96-well plastic plates or in 24-well plates containing glass poly-L-lysine-coated glass coverslips. In protein overexpression experiments, cells were transfected with expression plasmids 24 h after plating using Fugene 6 (Promega), following the manufacturer's protocol, with a 3:1 of Fugene/DNA ratio. For imaging analyses, transfected cells growing on glass coverslips were fixed for 15–30 min with 4% formaldehyde (Thermo Scientific) in phosphate-buffered saline (PBS) 48–72 h after transfection, rinsed with PBS and mounted on microscopy slides using the Vectashield (Vector Laboratories) mounting medium. Cells overexpressing GFP-tagged proteins were then imaged using and Olympus FV300 laser confocal microscope. Fluoview (Olympus) and Photoshop (Adobe) software were used for image display and analysis.

### Analysis of protein localization and aggregation

Protein localization and aggregation were quantified on confocal fluorescence images of HEK293 cells overexpressing GFP-tagged wt and mutant forms of full-length RUNX2, 48–72 h after transfection. The localization of the protein was assessed as being prevalently nuclear or cytoplasmic depending on the relative intensity of the diffuse fluorescence of the nucleus and of the cytoplasm in single confocal sections encompassing both cell compartments. In some cases, cells overexpressing certain RUNX2 mutants still displayed a substantially nuclear diffuse fluorescence but also some cytoplasmic and/or nuclear aggregates. These cells were assessed as having a prevalently nuclear localization of the protein. The presence of aggregates was determined by analyzing both single confocal sections and *z*-stack projections. Protein localization and aggregation were quantified for wt and mutant forms of RUNX2 by analyzing multiple 350 × 350 µm microscopy fields (*n* ≥ 10) from at least three different coverslips of cells transfected in distinct experiments. At least 500 cells were counted for each overexpressed construct. The occurrence of protein aggregation in cells was quantified in confocal images of HEK293 cell overexpressing GFP-tagged RUNX2-ccd, ATX7-ccd, their CC-stabilized mutants, and ccA7, ccAA, ccA21, ccAP and ccAL, 48 h after transfection owing to the faster aggregation kinetics of these peptides. Protein aggregation was quantified by analyzing multiple 175 × 175 µm microscopy fields (*n* = 8–16) from at least three different coverslips of cells transfected in distinct experiments. For each overexpressed construct, 150–350 cells were counted.

### Ultracentrifugation and dot immunoblotting assay

To assay the aggregation state in cells of wt and mutant forms of RUNX2, HEK293 cells overexpressing the different GFP-tagged RUNX2 forms were lysed 72 h after transfection in lysis buffer (10 mm Tris, pH 7.5, 100 mm NaCl, 0.5% sodium deoxycholate and 0.5% Nonidet P-40, containing the protease inhibitors pepstatin and leupeptin (1 mg/ml), 0.5 mm phenylmethylsulfonyl fluoride and 2 mm EDTA), and the lysates were centrifuged at 20 000*g* for 30 min, as previously described (15). After centrifugation, supernatant and pellet fractions were separated, and pellets were resuspended in lysis buffer. Equal volumes of supernatant and pellet fractions were then dot-immunoblotted on nitrocellulose membranes. Membranes were probed with a primary monoclonal anti-GFP antibody (Novus Biologicals) and with an anti-mouse HRP-conjugated secondary antibody (Calbiochem). Antibody binding to the membranes was then revealed using the Pierce ECL Western Blotting Substrate (Thermo Scientific), and chemiluminescence was detected on film (GE Healthcare). Developed films were imaged using a digital scanner (Canon), and blot intensities were quantitated by densitometry using the ImageJ software (NIH). In each experiment, an aggregation index was calculated as the ratio between the blot intensities of pellet and supernatant fractions for each lysate, and values were then normalized to the ratio of the wt RUNX2-GFP sample. The results of 7–8 experiments were then averaged and analyzed statistically.

### Luciferase reporter assay

To assess the relative transcriptional activity of RUNX2 mutants with respect to wt RUNX2, we performed a transcription reporter assay (41). The 6xOSE2 promoter sequence, which contains multiple consensus sites for RUNX2 binding (41), was synthesized (GeneArt, Invitrogen) and subcloned into the HindIII restriction site of the pGL3-Basic expression vector (e1751; Promega) directly upstream of the firefly luciferase coding sequence. This pGL3-6xOSE2-Luciferase plasmid was then co-transfected in HEK293 cells together with pcDNA6.2/C-EmGFP-DEST expression vectors encoding wt or mutant RUNX2 forms, and with a pRL-CMV vector (e2261; Promega) encoding for Renilla luciferase under a CMV promoter, which was used as a transfection control. The assessment of firefly luciferase expression driven by the 6xOSE2 promoter was performed 48–72 h after transfection by measuring sequentially the firefly and Renilla luciferase luminescence of cell lysates using the Dual-Glo luciferase assay system (e2920; Promega) following the manufacturer's procedure. Firefly luciferase luminescence values for each lysate were normalized against the corresponding Renilla luciferase luminescence values. We also performed control experiments in which the RUNX2-encoding plasmids and pRL-CMV were co-transfected with pGL3-Basic without the 6xOSE2 promoter, to measure background transcription. The normalized luminescence values of these promoter-less experiments were then subtracted from the luminescence values of the corresponding experiments that were performed with the 6xOSE2 promoter. In some luciferase experiments, the pcDNA6.2/V5-DEST plasmid was used to drive the expression of wt and mutant RUNX2 forms instead of the pcDNA6.2/C-EmGFP-DEST vector. Similar results were obtained with the two expression plasmids, and the data obtained with these constructs were pooled and analyzed statistically.

### MTT-formazan toxicity assay

Cell viability in HEK293 cultures was assessed after 72–96 h of overexpression of RUNX2-GFP or of its mutants through a colorimetric reaction, by measuring the cellular reduction of 3-(4,5-Dimethylthiazol-2-yl)-2,5-Diphenyltetrazolium Bromide (MTT; Molecular Probes-Invitrogen) to formazan (44). To this aim, cells grown in 96-well plates were incubated for 2–4 h at 37°C with 0.4 mg/ml MTT. After removal of the culture medium, the formazan precipitate that had formed was solubilized by adding 0.04 N HCl in isopropanol. The solubilized formazan generated a purple coloration that was quantified using a Glomax-Multi detection system (Promega) measuring absorbance at 560 nm.

### Statistics

Data are expressed as mean ± standard error of mean (SEM). Statistical analyses were performed using SPSS (IBM) and Excel (Microsoft) software. Chi-squared (χ^2^) with Yates' correction, Pearson's *r* correlation test, Student's *t*-test and one-way ANOVA, followed by *post hoc* tests, were used where appropriate as indicated in the section Results. In all instances, differences were considered as statistically significant when *P* < 0.05.

## SUPPLEMENTARY MATERIAL

Supplementary Material is available at *HMG* online.

## FUNDING

This research was supported by Telethon Italia (grant GGP11223). Funding to pay the Open Access publication charges for this article was provided by Telethon Italia.

## Supplementary Material

Supplementary Data
