# Supplementary material for: Association of polyalanine and polyglutamine coiled coils mediates expansion disease-related protein aggregation and dysfunction
Source: Hum Mol Genet. 2014 Feb 4;23(13):3402–20. doi: 10.1093/hmg/ddu049 (PMC4049302; doi:10.1093/hmg/ddu049)
Supplement: Supplementary Data [file supp_23_13_3402__index.html]

Association of polyalanine and polyglutamine coiled coils mediates expansion disease-related protein aggregation and dysfunction — Association of polyalanine and polyglutamine coiled coils mediates expansion disease-related protein aggregation and dysfunction — Association of polyalanine and polyglutamine coiled coils mediates expansion disease-related protein aggregation and dysfunction — Supplementary Data 

# Association of polyalanine and polyglutamine coiled coils mediates expansion disease-related protein aggregation and dysfunction

## Supplementary Data

Supplementary Data

**Files in this Data Supplement:**

- Supplementary Data - Pdf file
