# Supplementary material for: Association of polyalanine and polyglutamine coiled coils mediates expansion disease-related protein aggregation and dysfunction
Source: Hum Mol Genet. 2014 Feb 4;23(13):3402–20. doi: 10.1093/hmg/ddu049 (PMC4049302; doi:10.1093/hmg/ddu049)
Supplement: Supplementary Data [file supp_ddu049_ddu049supp.pdf]

SUPPLEMENTAL MATERIAL

Supplemental table 1 – *Number of proteins containing Q4, A4, QA4 repeats, their associations, and CC domains in each of the six proteomes analyzed.*

Species	Proteome	Q4	A4	QA4	Q4+A4	Q4+QA4	A4+QA4	Q4+A4+QA4	CC
Homo sapiens	20240	464	1565	278	110	25	51	13	4141
Mus musculus	16446	390	1194	202	83	20	29	8	3503
Xenopus tropicalis	23484	446	782	147	55	14	14	5	4942
Drosophila melanogaster	17515	2210	2129	285	979	139	126	96	3785
Caenorhabditis elegans	23514	656	852	143	156	58	50	37	3712
Saccharomyces cerevisiae	6619	235	137	35	15	11	3	2	1247

Supplemental table 2 – *Number of proteins containing associations of CC domains domains with Q4, A4, QA4 repeats in each of the six proteome analyzed.*

Species	Proteome	Q4 +CC	A4 +CC	QA4 +CC	Q4+A4 +CC	Q4+QA4 +CC	A4+QA4 +CC	Q4+A4+QA4 +CC
Homo sapiens	20240	211	419	120	52	15	31	8
Mus musculus	16446	177	347	87	38	13	16	4
Xenopus tropicalis	23484	208	244	61	27	11	7	4
Drosophila melanogaster	17515	902	792	139	446	79	75	58
Caenorhabditis elegans	23514	250	302	66	77	35	30	27
Saccharomyces cerevisiae	6619	103	32	18	8	9	2	2

LEGENDS TO SUPPLEMENTAL FIGURES

Supplemental Figure 1 – *Philogenetically-graded association and CC propensity of polyQ, polyA, and polyQA repeats*

A. Correlation of the %QA4/%Q4 ratio (*left panel*; Pearson's r = -0.92, p<0.01, R^2=0.85) or of the %A4/%QA4 ratio (*right panel*; Pearson's r = -0.23, p=0.65, with a R^2=0.05) in *Homo* (H), *Mus* (M), *Xenopus* (X), *Drosophila* (D), *Caenorhabditis* (C), and *Saccharomyces* (S) with phylogenetic distances, estimated as the time of evolutionary divergence of each species from *Homo sapiens* expressed in millions of years (*mya*). Note how the ratio between the percentages of QA4 and Q4 proteins in each proteome gradually increases from *Saccharomyces* to *Homo*, similar to what was observed for the %A4/%Q4 ratio. Conversely (*right panel*), the ratio between the percentage of polyA and polyQA proteins shows a quite stable value around 6 along the

phylogenetic scale, indicating that the occurrences of polyA and polyQA proteins tend to co-vary throughout phylogenesis. **B.** Histograms showing the observed and expected occurrence in proteomes of Q4+A4 (*left* panel), Q4+QA4 (*middle* panel), A4+QA4 (*right* panel). Values are normalized to the expected occurrence in each species. **C.** Correlation of the percent occurrence of Q4+A4 proteins in *Homo* (H), *Mus* (M), *Xenopus* (X), *Drosophila* (D), *Caenorhabditis* (C), and *Saccharomyces* (S) with phylogenetic distances, estimated as the time of evolutionary divergence of each species from *Homo sapiens* expressed in millions of years (*mya*). **D.** Examples of variably intermixed polyQ, polyA, and polyQA CC-prone stretches. The graphs show the per-residue CC prediction for the human proteins RL4 and ZN384, the *Drosophila* protein G32062, and the *Caenorhabditis* protein PQN-41. Regions with high CC propensity, repeats and primary sequences above the plots are labeled as in Fig. 1G. QA repeats are *in yellow*. *Grey* numbers indicate the position of the adjacent amino acid in the primary sequence. **E.** Modified helical nets of CC regions of human RUNX2, and *Drosophila* polyhomeotic distal (PHOD) and spalt-major (SALM) proteins. Note how in RUNX2 the polyQ and polyA stretches are flanked by valines in positions *a/d* and are separated by a single glutamate in position *e*. In the *Drosophila* protein PHOD, short polyQ and polyQA stretches are separated by CC-stabilizing hydrophobic residues, resembling a valine zipper (28), while longer polyA stretches alternate with canonical heptads in the protein SALM. Q, A, and QA repeats of at least four residues are labeled in *red*, *green*, and *yellow*, respectively, and marked by colored bars on the sides. *Yellow* boxes mark canonical hydrophobic residues in *a/d* positions. *Grey* boxes mark Q/A residues in *a/d*. *Black dots* are placed above charged residues in *e/g* positions.

Supplemental Figure 2 – *Biophysical and biochemical characterization of polyQ, polyA, and mixed peptides*

A. CD spectra of some of the peptides shown in Fig. 2A, dissolved in benign buffer, pH 7.4. Spectra were collected at different temperatures, starting from 5 °C (*black* traces), to 25 °C (*dark grey* traces) and 50 °C (*medium grey* traces), up to 75 °C (*light grey* traces). **B.** CD spectra of peptides ccAL (*left* panel) and ccA21 (*middle* panel) measured at 25 °C in benign buffer (*black* traces) or in the same buffer with 50% TFE (*gray* traces). The *right* panel shows the quantification of the 222/208 nm ellipticity ratio of the CD spectra shown in the *left* and *middle* panels. TFE causes the ratio to fall below 1 for both peptides. **C.** Quantification of the 222 nm ellipticity as a function of temperature in the 5-75 °C range of RUNX2-ccd and RUNX2(cc+)-ccd peptides. For each peptide, values are normalized to the 222 nm ellipticity measured at 5 °C. **D.** CD spectrum of the peptide RUNX2(cc+)-ccd measured at 5 °C in benign buffer with 50% TFE.

TFE causes the 222/208 nm ellipticity ratio to fall below 1. **E.** Silver-stained tricine gels as in Fig. 2E-F. To better highlight more subtle differences between ccAA, ccQQ, and the hybrid ccA/QA and ccQ/A peptides, the cross-linking reaction was performed at 15 °C (see *Methods* section).

Supplemental Figure 3 – *CC propensity and aggregation of* wt *and mutant forms of RUNX2*

A. CC propensity of some of the RUNX2 mutants shown in Fig. 3A as determined by Paircoil2, expressed as 1 minus the P-score assigned to each amino acid in the primary sequence. Protein segments whose CC propensity is 0.8-1 are highlighted in black. For simplicity, only the prediction for the N-terminal part of RUNX2 is shown. The site and effect on CC propensity of the different polyA length variations (partial deletion, *del*; expansion, *exp*) are highlighted by grey *arrowheads*. *Horizontal* arrowheads indicate deletion or expansions of the predicted CC domain related to corresponding variations in polyA length. **B**. Confocal fluorescence imaging of HEK293 cells overexpressing for 72 h GFP-tagged RUNX2(cc+/#1). The *left* panel shows a representative 100x100 µm field, the *right* panel shows a magnification of one representative cell. *Asterisks* indicate cell nuclei. *Arrows* indicate cells with a cytoplasmic mislocalization of the overexpressed protein. *Arrowheads* indicate intracellular aggregates.

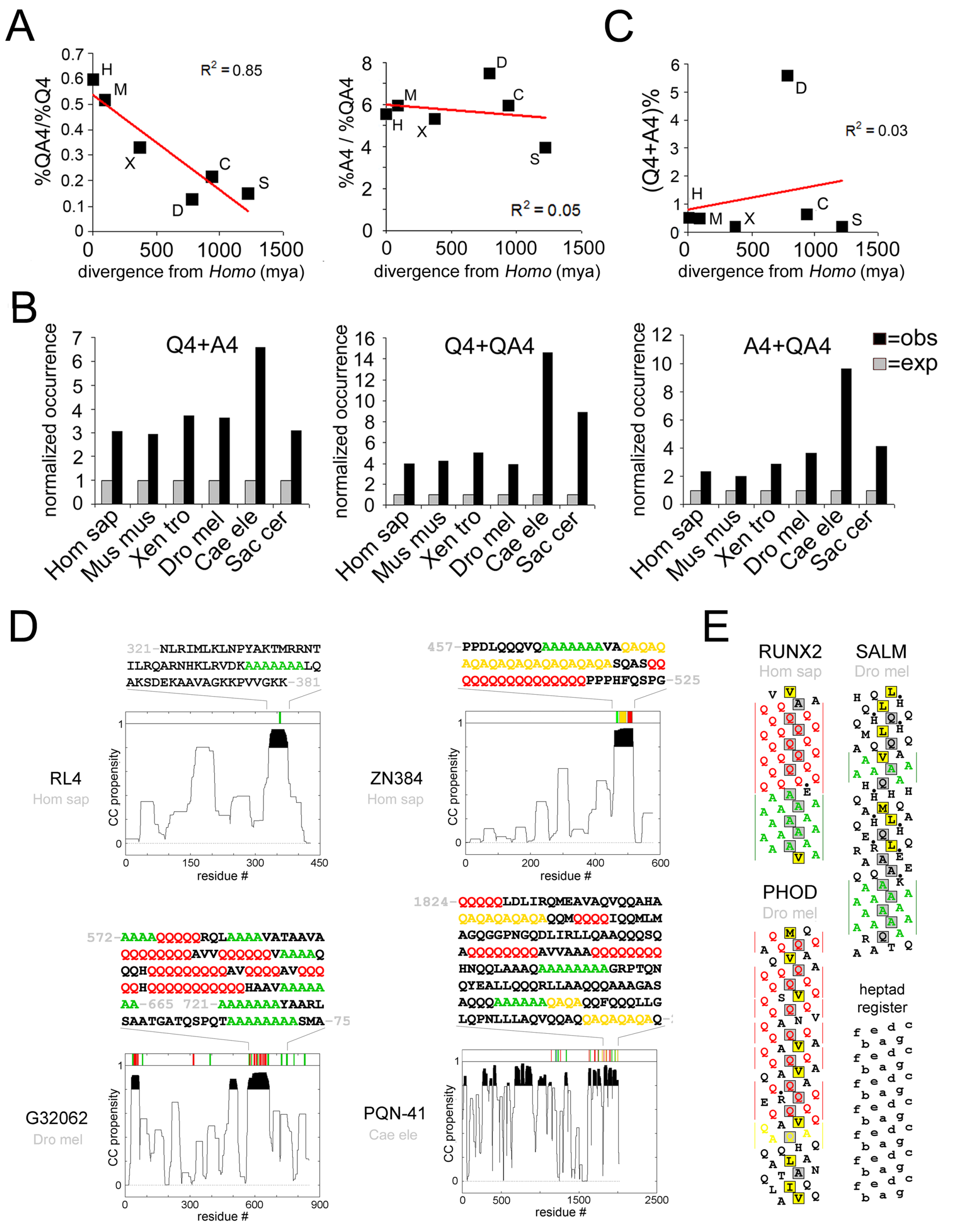

Supplemental Figure 1

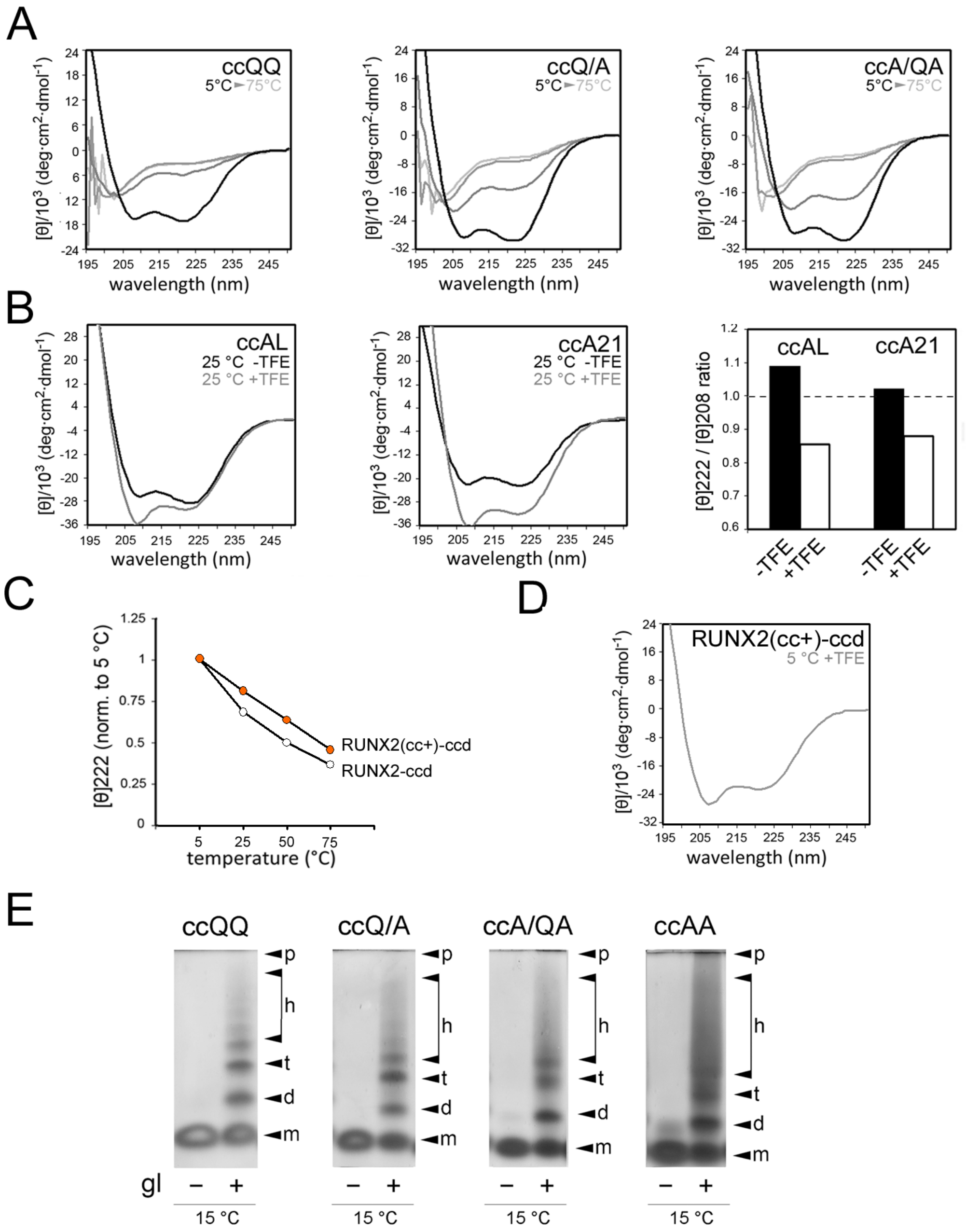

Supplemental Figure 2

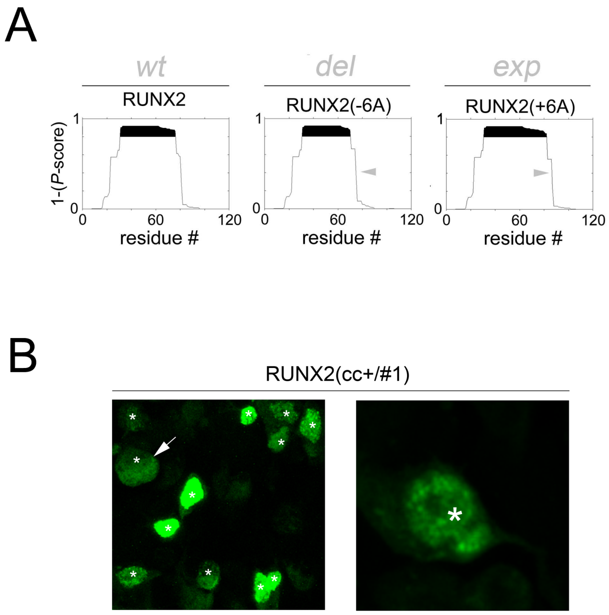

Supplemental Figure 3
